# Topical ferumoxytol nanoparticles disrupt biofilms and prevent tooth decay in vivo via intrinsic catalytic activity

**DOI:** 10.1038/s41467-018-05342-x

**Published:** 2018-07-31

**Authors:** Yuan Liu, Pratap C. Naha, Geelsu Hwang, Dongyeop Kim, Yue Huang, Aurea Simon-Soro, Hoi-In Jung, Zhi Ren, Yong Li, Sarah Gubara, Faizan Alawi, Domenick Zero, Anderson T. Hara, David P. Cormode, Hyun Koo

**Affiliations:** 1 Biofilm Research Labs, Levy Center for Oral Health, School of Dental Medicine, University of Pennsylvania, Philadelphia, PA 19104 USA; 2 Department of Orthodontics and Divisions of Pediatric Dentistry & Community Oral Health, School of Dental Medicine, University of Pennsylvania, Philadelphia, PA 19104 USA; 3 Department of Radiology, Perelman School of Medicine, University of Pennsylvania, Philadelphia, PA 19104 USA; 4 Department of Pathology, School of Dental Medicine, University of Pennsylvania, Philadelphia, PA 19104 USA; 5 Department of Cariology, Operative Dentistry and Dental Public Health, Oral Health Research Institute, Indiana University School of Dentistry, Indianapolis, IN 46202 USA; 6 Department of Bioengineering, School of Engineering and Applied Sciences, University of Pennsylvania, Philadelphia, PA 19104 USA

## Abstract

Ferumoxytol is a nanoparticle formulation approved by the U.S. Food and Drug Administration for systemic use to treat iron deficiency. Here, we show that, in addition, ferumoxytol disrupts intractable oral biofilms and prevents tooth decay (dental caries) via intrinsic peroxidase-like activity. Ferumoxytol binds within the biofilm ultrastructure and generates free radicals from hydrogen peroxide (H_2_O_2_), causing in situ bacterial death via cell membrane disruption and extracellular polymeric substances matrix degradation. In combination with low concentrations of H_2_O_2_, ferumoxytol inhibits biofilm accumulation on natural teeth in a human-derived ex vivo biofilm model, and prevents acid damage of the mineralized tissue. Topical oral treatment with ferumoxytol and H_2_O_2_ suppresses the development of dental caries in vivo, preventing the onset of severe tooth decay (cavities) in a rodent model of the disease. Microbiome and histological analyses show no adverse effects on oral microbiota diversity, and gingival and mucosal tissues. Our results reveal a new biomedical application for ferumoxytol as topical treatment of a prevalent and costly biofilm-induced oral disease.

## Abstract (web summary)

Ferumoxytol is a nanoparticle formulation approved for systemic use to treat iron deficiency. Liu et al. show that topical use of ferumoxytol, in combination with low concentrations of H_2_O_2_, disrupts intractable oral biofilms and prevents tooth decay in vitro and in an animal model.

## Introduction

One of the first nanoparticle formulations to be Food and Drug Administration (FDA)-approved for clinical use was an iron oxide nanoparticle contrast agent for magnetic resonance imaging (Feridex), while another similar preparation (ferumoxytol) was subsequently approved for treatment of iron deficiency^1–3^. Recently, additional biomedical applications have started to emerge for experimental iron oxide nanoparticles, including tumor prevention and biofilm disruption^4,5^. Most human infections are caused by microbial biofilms that are notoriously challenging to remove or treat because the microorganisms are embedded in a protective matrix of extracellular polymeric substances, such as exopolysaccharides (EPS)^6,7^. The matrix reduces drug access, triggers bacterial drug tolerance, while enhancing the mechanical stability of the biofilm^6,7^. Therefore, more effective antiinfective therapies will need to target the biofilm matrix, as well as the individual microbial cells within.

Dental caries is a classic biofilm-induced disease that causes the destruction of the mineralized tooth tissue^8^. It remains the most prevalent human health condition affecting 3.5 billion people globally, mostly underprivileged children and families, costing >$120 billion in the USA alone^9,10^. In particular, severe childhood tooth decay is often associated with iron deficiency anemia, posing a serious public health challenge^10–13^. The disease-causing biofilms develop when pathogens such as *Streptococcus mutans* and other cariogenic bacteria assemble an extracellular matrix rich in EPS and acidify the biofilm microenvironment^14^. The bacteria embedded in the biofilm matrix produce highly acidic microenvironments with pH values close to 4.5, which erode the tooth apatite leading to the onset of dental caries. Current antimicrobials and clinical modalities are incapable of degrading EPS, and have limited killing activity against biofilm cells^7^. New therapeutic strategies that could activate substances in situ to target the vital structural and biological traits of biofilms, e.g., the matrix and embedded cells in acidic pH conditions may lead to localized effects without damaging the surrounding tissues.

Experimental iron oxide nanoparticles have been shown to exhibit an intriguing enzyme mimetic activity^5,15,16^. These catalytic nanoparticles have high peroxidase-like activity at pathogenic acidic pH values, thereby locally activating free-radical generation from hydrogen peroxide (H_2_O_2_) to provide antibiofilm effects^17^. Thus, the catalytic properties of nanoparticles can be exploited to achieve a more focused and biofilm-specific antiinfective therapy. However, the potential for catalytic-therapeutic action has been largely assumed to be absent when formulated for medical use (e.g., ferumoxytol) due to passivating coatings^5^. Interestingly, a recent study reported that ferumoxytol nanoparticles, administered systemically, inhibited tumor growth in mice by enhancing the production of macrophage associated reactive oxygen species^4^.

Here, we show that ferumoxytol can display intrinsic peroxidase-like properties in a pH-dependent manner, be retained within biofilm following topical treatment, and provide localized catalytic activity to prevent a costly and prevalent oral disease in vivo. Time-lapsed studies reveal that ferumoxytol nanoparticles efficiently catalyze H_2_O_2_ under acidic condition for simultaneous bacterial killing and breakdown of EPS structure. The nanoparticles bind within the biofilm ultrastructure, causing bacterial membrane damage and polymer matrix degradation in situ upon exposure to low concentrations of H_2_O_2_. Using ex vivo biofilm and rodent models of severe early childhood caries, we find that topically applied ferumoxytol effectively suppresses biofilm accumulation and acid damage of the enamel surface, thereby preventing the onset of tooth cavitation without impacting the surrounding mucosal tissues and oral microbiota in vivo. Considering that ferumoxytol has been used off-label for treatment of iron deficiency in pediatric population^18^, its use against biofilms could be directly clinically applicable as a novel topical agent to prevent childhood dental caries.

## Results

### Catalytic properties and in vitro bioactivity of ferumoxytol

**Fig. 1:**
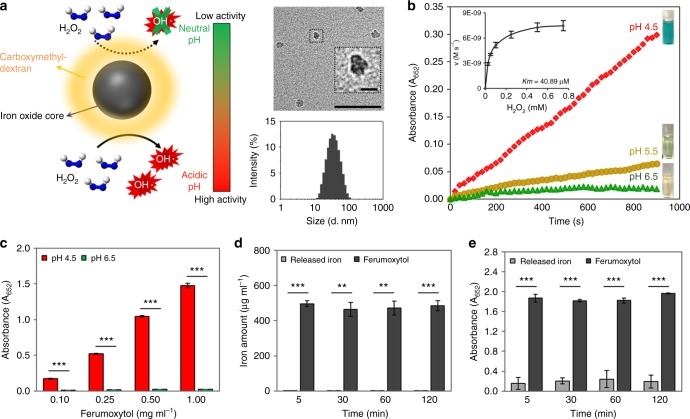
Characterization of catalytic properties and bioactivity of ferumoxytol. **a** Schematic depiction of the pH-dependent catalytic activity of ferumoxytol. Insets: negative stain TEM of ferumoxytol (Scale bar: 50 nm and 10 nm for close up image) and hydrodynamic diameter measurements. **b** Peroxidase-like activity of ferumoxytol at different pH values as determined by a colorimetric assay using 3,3’,5,5′-tetramethylbenzidine (TMB) and Michaelis–Menten kinetics plotting (inset). The catalytic reaction of TMB (which serves as a peroxidase substrate) in the presence of H_2_O_2_ produces a blue color. **c** Peroxidase-like activity of ferumoxytol at different concentrations at pH 4.5 and pH 6.5. **d** The amount of intact ferumoxytol and released free irons at pH 4.5 and their respective catalytic activities (**e**). The data are presented as the mean ± s.d. from three independent experiments (*n* = 6). The quantitative data were subjected to Student’s *t* test for a pairwise comparison. ^**^P < 0.01, ^***^P < 0.001

Ferumoxytol is a nanoparticle comprised of iron oxide cores coated with carboxymethyl-dextran. Transmission electron microscopy (TEM) shows the cores of ferumoxytol to be somewhat amorphous and 7.15 ± 0.95 nm in diameter (Fig. 1a). The negative staining indicated that the coating was 1.71 ± 0.47 nm thick. It has a hydrodynamic diameter of 23.0 ± 0.7 nm as determined by dynamic light scattering (Fig. 1a). In addition, we determined that for every 1 mg of iron in ferumoxytol, there is 1.06 ± 0.07 mg of carboxymethyl-dextran (Supplementary Fig. 1). The enzyme-like properties of ferumoxytol are illustrated in Fig. 1b–e. Ferumoxytol can rapidly catalyze H_2_O_2_, displaying peroxidase-like functionality in a pH-dependent manner as determined by a colorimetric method using 3,3′,5,5′-tetramethylbenzidine (TMB). The hydroxyl radicals produced from H_2_O_2_ can oxidize colorless TMB (which serves as a peroxidase substrate) to blue colored reaction products whose concentration can be assayed by measuring the absorbance at 652 nm^16^.

To determine the effect of pH on this observed peroxidase-like activity, ferumoxytol nanoparticles were incubated with TMB at several pH values (4.5–6.5). The absorbance of these solutions upon the addition of H_2_O_2_ is plotted against time in Fig. 1b, and photographs in the inset of Fig. 1b shows the color changes in each condition. Clearly, the ferumoxytol catalytic action is high at acidic pH (4.5), but is minimal at pH 6.5 (Fig. 1b). Michaelis–Menten steady-state kinetics of ferumoxytol iron nanoparticles were also determined (Fig. 1b inset). The data were plotted against the corresponding concentrations of H_2_O_2_ and fitted to a Michaelis–Menten curve. The *Km* value of ferumoxytol for H_2_O_2_ is 40.89 µM, indicating strong catalytic property (peroxidase-like activity), which is several folds better than that for horseradish peroxidase (HRP)^16^, suggesting that ferumoxytol has a much higher affinity for H_2_O_2_ than HRP. Next, we studied the peroxidase-like activity of ferumoxytol at different concentrations (Fig. 1c). We found that ferumoxytol highly catalyzes the reaction of the substrate in the presence of H_2_O_2_ for each concentration (0.1–1 mg ml^−1^) showing increasing performance with concentration. In addition, the catalytic activity of ferumoxytol at pH 6.5 is negligible compared to acidic pH at all concentrations tested.

We also examined whether the activation of H_2_O_2_ by ferumoxytol is due to catalytic activity from nanoparticles themselves or from released iron ions via the Fenton reaction. We found only trace amounts of free iron ions leached from ferumoxytol in acidic pH buffer (pH 4.5, Fig. 1d). Importantly, the catalytic activity of the solution phase is low (Fig. 1e), showing that the observed activity is primarily derived from the nanoparticle itself. Thus, ferumoxytol displays high catalytic activity despite the presence of carboxymethyl-dextran coating. To further understand the role of carboxymethyl-dextran, we performed experiments using iron oxide nanoparticles (IONP) of similar core size to ferumoxytol, that were coated with citrate, a labile coating molecule that should allow reagents access to the IONP surface. As expected, citrate-coated IONP had higher catalytic activity than ferumoxytol due to better access to the IONP surface^16^ (Supplementary Fig. 2). Carboxymethyl-dextran alone had negligible catalytic activity and its addition did not affect the activity of the citrate-coated IONP.

**Fig. 2:**
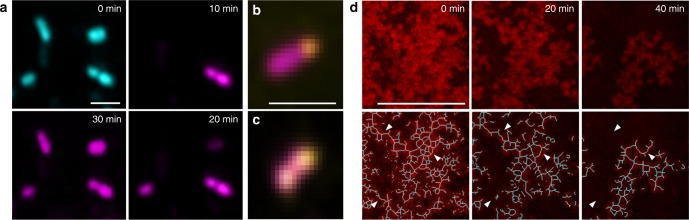
Time-lapsed bacterial killing and glucan degradation by ferumoxytol-activated H_2_O_2_. **a** Bacterial cells are labelled in blue by SYTO 60 and dead cells in purple by propidium iodide (*n* = 4). High-magnification close-up views of (**b**, **c**) single bacterial cell showing nanoparticles (labelled with Alexa Fluor 488, in yellow; upper/lower panels) bound to its surface (Scale bar: 1 µm). **d** Time-lapsed EPS glucans (labelled in red with Alexa Fluor 647-dextran conjugate) degradation by ferumoxytol-mediated H_2_O_2_ catalysis (*n* = 4) (Scale bar: 100 µm)

The ferumoxytol activation of H_2_O_2_ indicated that it could, therefore, function as a bacterial killing and EPS degrading system for targeting the acidic biofilm microenvironment. To assess the bioactivity of ferumoxytol-mediated H_2_O_2_ catalysis, we conducted high-resolution time-lapsed imaging using fluorescently labelled bacterial cells (*Streptococcus mutans*) and insoluble α-glucans (Fig. 2). Ferumoxytol (1 mg ml^−1^) was added to an actively growing bacterial cells suspension followed by exposure to H_2_O_2_ at a concentration of 1%, under acidic (4.5) or near neutral pH (6.5) conditions. To visually observe the distribution of viable and dead bacteria, intact *S. mutans* cells were labelled with SYTO 60 and propidium iodide (PI) was used to determine bacterial killing over time at the single-cell level. The fluorescence images show that *S. mutans* viability was affected as early as 10 min by ferumoxytol in the presence of H_2_O_2_ at pH 4.5. Bacterial cells are labelled in blue by SYTO 60 and the purple color indicates dead cells labelled by PI, a cell-impermeant molecule that can only enter cells with damaged membranes, rapidly gaining intracellular access following treatment (Fig. 2a). Conversely, *S. mutans* cells remained mostly viable at pH 6.5 (Supplementary Fig. 3a). In addition, close-up views of individual bacterial cells with high magnification show ferumoxytol nanoparticles (labelled with Alexa Fluor 488, in yellow) located on the cell surface (Fig. 2b, c).

Insoluble glucans are key virulence factors as they form the core of the extracellular matrix in cariogenic biofilms^14^. Thus, we also assessed whether these EPS can be broken down following incubation with ferumoxytol and H_2_O_2_. The EPS were labelled by Alexa Fluor 647-dextran conjugate during glucan synthesis as detailed previously^19^, allowing structure visualization and degradation monitoring via time-lapse imaging. As shown in Fig. 2d, the glucans (in red) were readily degraded when exposed to ferumoxytol and H_2_O_2_ in acidic pH. To further understand the EPS breakdown process over time, we applied computational analysis that generates geometrical scaffolds based on connectivity, topology, and length of the glucan structure. Intact glucans show a web-like structure, forming a meshwork of interwoven “EPS filaments” (Fig. 2d, blue lines). However, after exposure to ferumoxytol and H_2_O_2_, we observed gradual dismantling of the matrix structure by degrading the interconnected branches (see white arrowheads), resulting in a smaller EPS core with most of the shorter fragments completely degraded after 40 min (Fig. 2d). Comparatively, EPS glucans remained structurally intact when incubated with ferumoxytol and H_2_O_2_ at pH 6.5 (Supplementary Fig. 3b). These results indicate efficient bacterial killing with EPS degrading capabilities when ferumoxytol-mediated H_2_O_2_ catalysis is triggered at acidic pH.

### Antibiofilm activity of ferumoxytol in vitro

Localized biological efficacy requires retention of ferumoxytol within biofilm structures and bioactivity in pathological (acidic) microenvironment. *S. mutans*, an established biofilm-forming, acidogenic, and EPS-producing oral pathogen^14^, was used to form biofilms on saliva-coated hydroxyapatite (sHA) surfaces (tooth enamel-like materials) (Supplementary Fig. 4a). The retention and catalytic activity of ferumoxytol within biofilms were determined following topical treatments. To mimic a pathogenic situation, biofilms were formed in the presence of sucrose, which provides a substrate for EPS synthesis and acid production. The pH values in our model reach ~4.5, congruent with human plaque-biofilm pH at sites of active caries^20,21^. We used this model to determine both the amounts of ferumoxytol bound to biofilms and their peroxidase-like activity in situ.

**Fig. 3:**
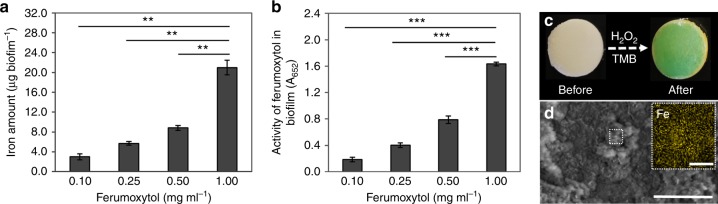
Ferumoxytol binding to the biofilm and in situ catalytic activity. **a** Amount of ferumoxytol bound and **b** catalytic activity within biofilms. **c** Photographic images of ferumoxytol treated biofilm before and after exposure to H_2_O_2_ in the presence of TMB (the blue color indicates free-radical generation via H_2_O_2_ catalysis in situ). **d** SEM image of ferumoxytol treated biofilm (Scale bar: 200 µm) and EDS-mapping image showing iron ions (yellow) distribution on selected area (Scale bar: 10 µm). The data are presented as the mean ± s.d. from three independent experiments (*n* = 6). The quantitative data were subjected to Student’s *t* test for a pairwise comparison. ^**^P *<* 0.01, ^***^P < 0.001

Quantitative analysis via inductively coupled plasma optical emission spectrometry (ICP-OES) shows that the amount of ferumoxytol bound to biofilms increased in a dose-dependent manner (Fig. 3a). Furthermore, we examined whether the nanoparticles bound within biofilms are catalytically active. Consistent with the amount of ferumoxytol adsorbed within the biofilm, the highest catalytic activity was achieved at concentration 1 mg ml^−1^ under the conditions tested (Fig. 3b). The representative photograph in Fig. 3c shows the color change caused by TMB oxidation (blue) in a biofilm treated with ferumoxytol 30 min after H_2_O_2_ addition, similarly to the nanoparticles in solution. Moreover, scanning electron microscopy with corresponding elemental mapping was conducted for analysis of ferumoxytol content within biofilm structures (Fig. 3d). These data show that Fe element is distributed throughout the biofilm structure, indicating the effective binding of ferumoxytol. We have also examined biofilms grown without sucrose to assess ferumoxytol accumulation in nonpathogenic conditions. As expected, the resulting lack of EPS caused reduced biomass and negligible amounts of ferumoxytol binding and minimal catalytic activity (below detection level; Supplementary Fig. 5). In addition, we used citrate-coated IONP to examine whether the carboxymethyl-dextran coating of ferumoxytol would play a protective role to the iron oxide core to help maintain catalytic activity in the presence of the EPS matrix. We found that the activity of citrate-coated IONP mixed with carboxymethyl-dextran was better than that of citrate-coated IONP alone (Supplementary Fig. 6), suggesting a beneficial role of dextran-coating for the catalytic activity of the nanoparticle within the biofilm structure.

**Fig. 4:**
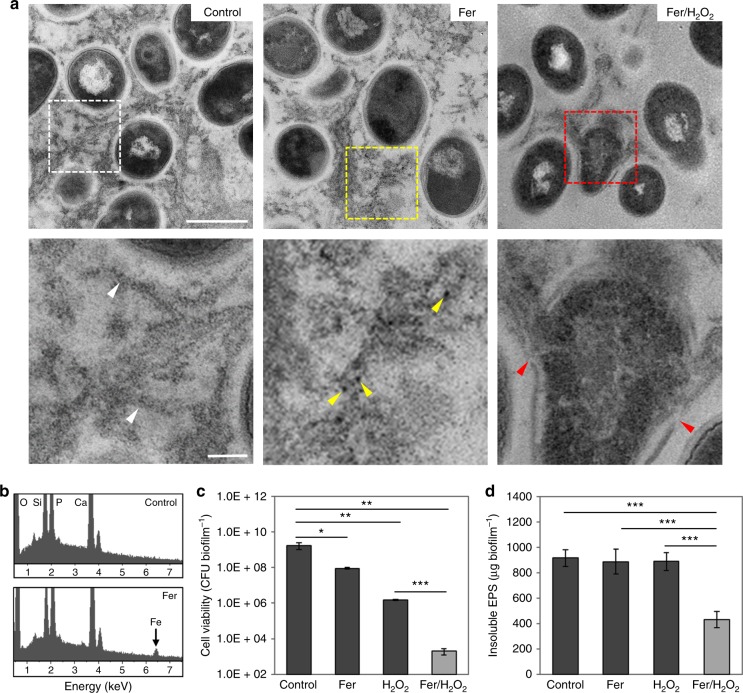
Antibiofilm performance of ferumoxytol/H_2_O_2_. **a** High resolution/magnification TEM images of untreated biofilm ultra-structure where bacterial cells can be seen embedded in EPS matrix (white box and white arrowheads); Ferumoxytol bound within biofilm (yellow box): higher magnification image shows electron-dense nanoparticles (yellow arrowheads) bound to EPS matrix; TEM of EPS degradation and bacterial morphological damage (red box and red arrowheads) (Scale bars: 500 nm for upper panel and 100 nm for lower panel). **b** EDS spectra of untreated and ferumoxytol-treated biofilms; **c** Effect on the viability of *S. mutans* cells within biofilms as well as EPS degradation (**d**) following exposure to ferumoxytol and/or H_2_O_2_. The data are presented as the mean ± s.d. from three independent experiments (*n* = 6). The quantitative data were subjected to Student’s *t* test for a pairwise comparison. ^*^P < 0.05, ^**^P *<* 0.01, ^***^P < 0.001

To examine ferumoxytol binding in situ, we employed TEM and energy dispersive spectroscopy (EDS) to visually observe and confirm the nanoparticles within the biofilm ultrastructure. Cross-sectional images of a control biofilm show abundant extracellular matrix (Fig. 4a: white box is a representative area of EPS) containing strands of fibrilar polysaccharides (white arrowheads) interspersed with amorphous substances surrounding individual bacterial cells similar to those observed in dental biofilms;^22^ no Fe signal is detected by EDS in control biofilms (Fig. 4b). In biofilms treated with ferumoxytol, we observed electron-dense nanoparticle-like structures associated with the extracellular matrix (Fig. 4a yellow box and yellow arrowheads). The existence of Fe signals is found in the EDS spectra of ferumoxytol-treated biofilm (Fig. 4b), consistent with ferumoxytol localization in the biofilms as determined by the elemental mapping (Fig. 3d), additionally illustrating the successful incorporation of ferumoxytol into biofilms following topical treatment.

Considering the effective ferumoxytol binding and catalytic activity within biofilms, we investigated whether the bound nanoparticles could catalyze H_2_O_2_ to breakdown the EPS matrix and kill the embedded bacteria in situ. TEM analysis of ferumoxytol-treated biofilms followed by H_2_O_2_ exposure reveals physical disruption of bacterial cell membranes and extrusion of the intracellular content (Fig. 4a, red box and red arrowheads). This is consistent with membrane damage caused by free radicals via lipid peroxidation^23,24^ and PI labelling of membrane-compromised *S. mutans* cells as observed via confocal imaging (Fig. 2a). Moreover, the matrices of the treated biofilms are highly degraded and devoid of structured and fibrilar polysaccharides, while mostly amorphous and scattered EPS were observed in the TEM images (Fig. 4a). In marked contrast, the biofilms treated with vehicle control or ferumoxytol alone were intact without significant disruption of either bacteria or EPS matrix (Fig. 4a).

To further confirm the bioactivity of ferumoxytol, the number of viable cells and EPS content were determined in the treated biofilms (Fig. 4c, d). The results show potent biocidal activity against *S. mutans* within biofilm, with >99.9% killing in 5 min (Fig. 4c). Consistent with biofilm binding and catalytic activity, ferumoxytol at 1 mg ml^−1^ was most effective with 1% H_2_O_2_ (Supplementary Fig. 4b–c, *P* *<* 0.01–0.001 by paired *t* test) causing >6-log reduction of viable cells compared to vehicle control and >1000-fold more effective than H_2_O_2_ alone (Fig. 4c, *P* *<* 0.01–0.001 by paired *t* test). Importantly, the treatment significantly reduced the amount of insoluble glucans compared to control and to H_2_O_2_ or ferumoxytol alone, further indicating the EPS-degrading capability of ferumoxytol-mediated H_2_O_2_ catalysis (Fig. 4d, *P* < 0.001 by paired *t* test). Horseradish peroxidase and glucanohydrolases were also tested as additional controls. As expected, peroxidase (at equivalent activity to ferumoxytol) catalyzed H_2_O_2_ to kill planktonic bacteria (Supplementary Fig. 7a), while glucanohydrolases digested insoluble glucans (Supplementary Fig. 7b) in our test system, thereby confirming that ferumoxytol-mediated H_2_O_2_ catalysis exhibit both antibacterial and EPS degradation properties. However, experiments performed in biofilms also highlighted the advantages of ferumoxytol, given the lack of antibacterial activity of glucanohydrolases^25^ and low efficacy of peroxidase in biofilms (Supplementary Fig. 8). The limited peroxidase bioactivity may be due to the poor stability of natural enzymes (vs. inorganic enzyme mimetics) when exogenously added in biological systems^5,15^. Altogether, the data indicate that ferumoxytol is retained in biofilms following topical exposure, and display pH-dependent catalysis of H_2_O_2_ in situ, which can enhance killing potency against the embedded bacteria and degrade the EPS matrix under acidic conditions.

### Disruption of ex vivo biofilms and human enamel demineralization

**Fig. 5:**
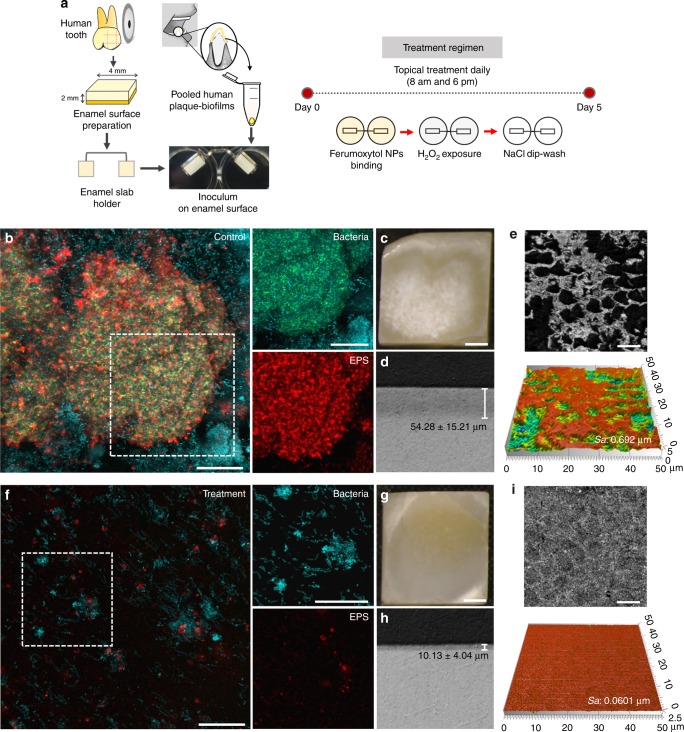
Antibiofilm properties of topical ferumoxytol/H_2_O_2_ treatments using an ex vivo biofilm model. **a** Experimental design and processing. **b**, Confocal imaging of the morphology of vehicle-control treated biofilm and **f** biofilm treated with ferumoxytol/H_2_O_2_ (white box indicates selected area for close-up images of bacteria and EPS components; Scale bar: 50 µm): total bacteria and *S. mutans* cells are labelled in blue and green, respectively; EPS are in red (Scale bar: 50 µm). **c**, Light microscopy images of the enamel surface of untreated biofilm showing “white spot-like caries lesions” and **g** ferumoxytol/H_2_O_2_ treated biofilm showing intact and smooth surface (Scale bar: 1 mm). **d**, **h** Lesion depth of the enamel surfaces (control and treated). **e**, **i** Representative confocal topography of enamel surfaces and enamel roughness (control and treated) (Scale bar: 10 µm). The data presented as mean ± s.d. from triplicates of two independent experiments (*n* = 6)

We next developed a combination therapy consisting of topical application of ferumoxytol (at 1 mg ml^−1^) immediately followed by H_2_O_2_ (at 1%) exposure, twice daily to simulate oral use. Using this treatment regimen, our preliminary studies show that topical ferumoxytol with H_2_O_2_ exposure strongly disrupted *S. mutans* biofilm accumulation on sHA surfaces (Supplementary Fig. 9, *P* < 0.001 by paired *t* test). To gain further insight into the therapeutic potential of our approach, we used an ex vivo human biofilm model to assess whether ferumoxytol/H_2_O_2_ can disrupt cariogenic biofilm and prevent enamel surface damage. In this model, plaque-biofilm samples were collected from diseased patients affected by severe childhood caries, and inoculated for biofilm development on natural human tooth-enamel (see diagram in Fig. 5a). The microscale spatial distribution and structural organization of the biofilm components were determined via a multilabeling approach using total bacteria and *S. mutans* specific fluorescent probes, with EPS-matrix labeling via an Alexa Fluor 647-dextran conjugate. The control human-derived biofilms had ‘dome-shaped’ bacterial clusters (in blue) spatially arranged with EPS (in red) matrix (Fig. 5b) that are typically found when grown under cariogenic conditions in the presence of sucrose. Cross-sectional confocal images reveal localized bacterial aggregates comprised mostly of *S. mutans* cells (in green, Fig. 5b bacteria panel) that are surrounded by an interconnected EPS-matrix (in red, Fig. 5b EPS panel) forming cohesive and densely packed microbial structure. In a sharp contrast, only small cell clusters with sparsely distributed EPS were detected in the ferumoxytol/H_2_O_2_ treatment group (Fig. 5f).

The striking differences in both bacterial density and structural organization could inflict differential damage of the mineralized tooth tissue underneath the distinctive biofilms. Macroscopically, we observed large areas of enamel surface demineralization in the control group; close-up views show chalky and white spot-like demineralization, similar to early caries lesions seen clinically (Fig. 5c). In contrast, the enamel surface from ferumoxytol/H_2_O_2_ treatment was essentially devoid of such opaque demineralized areas (Fig. 5g). The observed visual differences were confirmed with confocal topography imaging and transversal microradiography analysis. The enamel surfaces from the control group have eroded forming micro-cavities resulting in ~10 times higher surface roughness (*Sa* values) than that treated with ferumoxytol/H_2_O_2_, whose enamel surface was mostly intact and smooth (Fig. 5e, i). Importantly, the lesion depth as determined by microradiography is significantly deeper in the control group (vs. ferumoxytol/H_2_O_2_ group, Fig. 5d, h, *P* < 0.01 by paired *t* test). Collectively, these findings reveal that ferumoxytol-mediated H_2_O_2_ catalysis can potently disrupt the development of cariogenic biofilms and prevent localized demineralization and caries-like lesions on tooth-enamel surface.

### In vivo inhibition of dental caries

**Fig. 6:**
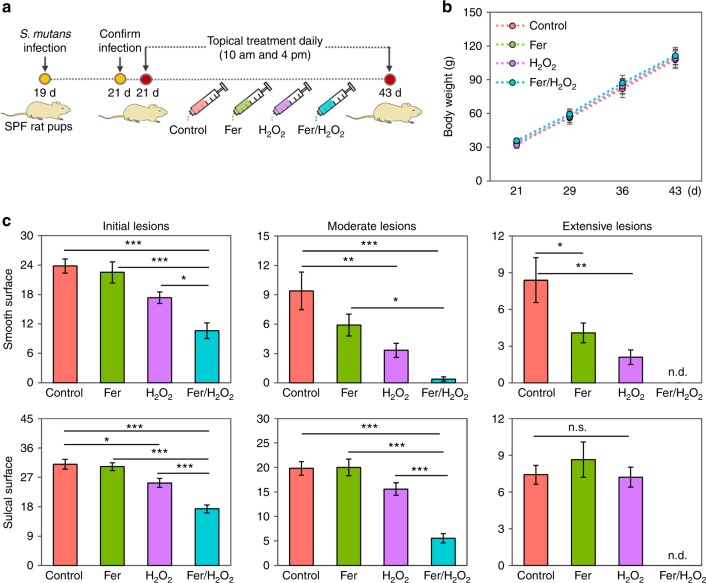
Therapeutic efficacy of topical ferumoxytol/H_2_O_2_ against a biofilm-associated oral disease (tooth decay) in vivo. In this model, tooth-enamel progressively develop caries lesions (analogous to those observed in humans), proceeding from initial areas of demineralization to moderate lesions and on to extensive (severe) lesions characterized by enamel structure damage and cavitation. **a** Experimental design and treatment regimen. **b** Body weights of rat pups during the experimental period. **c** Caries onset and severity of both smooth and sulcal surfaces. Caries scores were recorded as stages and extent of carious lesion severity according to Larson’s modification of Keyes’ scoring system^26,27^. The data presented as mean ± s.d. (*n* = 10), and one-way ANOVA with post hoc Tukey HSD test was used for a multiple comparison. ^*^P < 0.05, ^**^P < 0.01, ^***^P < 0.001; n.d. nondetectable; n.s. nonsignificant

We next sought to determine whether topical ferumoxytol/H_2_O_2_ treatment could suppress tooth decay using an established rodent model that mimics the characteristics of severe early childhood caries, including *S. mutans* infection of rat pups and protracted feeding of sugar-rich diet^26^. We simulated the conditions that might be experienced clinically in humans by applying the test agent solutions topically (orally delivered; 100 μl per animal) twice daily with a brief, 1min exposure time (Fig. 6a)^27^ to mimic the use of a mouthwash. Using this treatment regimen, we assessed the incidence and severity of caries lesions on teeth of rat pups. During the 3-week experimental period, the rats remained in apparent good health and no significant differences in body weights between control and all test groups were detected (Fig. 6b). Treatments with ferumoxytol/H_2_O_2_ resulted in potent suppression of caries development at all relevant sites (both smooth and sulcal surfaces).

Quantitative caries scoring analyses reveal that ferumoxytol/H_2_O_2_ greatly attenuated the initiation and severity of caries lesions (vs. vehicle control, Fig. 6c, *P* *<* 0.001 by one way ANOVA with post hoc Tukey HSD test), and completely blocked extensive enamel damage, preventing the onset of cavitation on both smooth and sulcal dental surfaces. Furthermore, the efficacy of combination therapy was significantly higher than H_2_O_2_ or ferumoxytol alone (*P* *<* 0.05–0.001 by one way ANOVA with post hoc Tukey HSD test), reducing more effectively the number and severity of caries lesions, supporting the catalytic-therapeutic mechanism of ferumoxytol activation of H_2_O_2_. We also observed proportionally greater effects on moderate and extensive carious lesions than on initial caries, which may be related to the conditions mimicking severe childhood caries. Considering the dynamics of caries development, it is possible that the effects on less severe lesions could have been observed at earlier time points. It is also noteworthy that ferumoxytol targets the biofilm and inclusion of agents that interfere with demineralization and enhance remineralization, such as fluoride, could enhance the therapeutic effects on initial carious lesions.

**Fig. 7:**
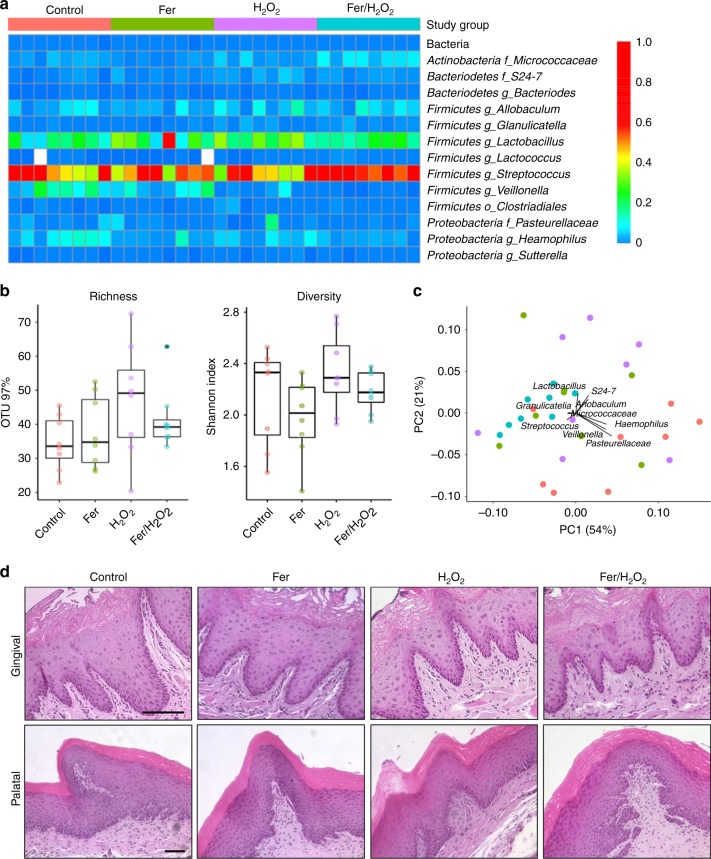
Effects of topical ferumoxytol/H_2_O_2_ on oral microbiome and soft tissue in vivo after 21 days of treatment. **a** The heatmap shows main bacterial genera found across all samples, distributed by treatment groups (*n* = 8, for each treatment group). **b** Richness and diversity show no significant differences among groups (*P* > 0.05 by Wilcoxon rank sum test). **c** Weighted Unifrac principal coordinate analysis (PCoA) revealed that the ferumoxytol/H_2_O_2_ group has similar composition and the lowest distances between samples (*P* *<* 0.001 by PERMANOVA test). **d** Histopathology of gingival and palatal tissue in animals treated with ferumoxytol/H_2_O_2_ is similar to control (Scale bar: 100 µm)

To evaluate the overall effects on oral microbiota and surrounding tissues after 21 days of topical treatment, the microbiome sequencing and histopathological images of soft oral tissues are presented in Fig. 7. All treatment groups showed similar oral microbial composition (Fig. 7a) with no significant differences in alpha diversity among each other (Fig. 7b; *P* > 0.05 by Wilcoxon rank sum test), suggesting our treatments did not disrupt the ecological balance of the microbiota. Interestingly, weighted Unifrac distances analyzed of principal coordinate analysis (PCoA) by treatment groups revealed that ferumoxytol/H_2_O_2_ group has similar composition with lowest dispersion (Fig. 7c, blue dots; *P* < 0.001 by PERMANOVA test) than other groups. Hematoxylin and eosin (H&E) images of gingival and palatal tissues from all experimental groups showed no visible signs of harmful effects, such as proliferative changes, inflammatory responses, or necrosis (Fig. 7d), indicating high histocompatibility of ferumoxytol/H_2_O_2_ treatment. Taken together, the data show that topical ferumoxytol/H_2_O_2_ treatments can efficiently suppress the development of a costly and prevalent oral disease without affecting the oral microbiota composition or showing deleterious effects in the surrounding soft-tissues in vivo.

## Discussion

Our data provide direct evidence of a new biomedical application for a FDA-approved nanoparticle formulation for systemic administration that can effectively disrupt biofilms and prevent severe tooth decay under a topical treatment regimen. We found an alternate catalytic-therapeutic mechanism governed by intrinsic “peroxidase-like” activity, which can be achieved with low doses of topical ferumoxytol with H_2_O_2_ applications. Importantly, this mechanism is pH-dependent so that the catalysis is activated under the specific acidic pH values found in cariogenic biofilms but attenuated at pH values close to neutral (physiological), avoiding unmitigated free radical production. Ferumoxytol retained within biofilms can locally activate H_2_O_2_ for in situ bacterial killing via membrane disruption and EPS matrix degradation through glucan structure cleavage. Such properties thwarted cariogenic biofilm accumulation and prevented enamel surface damage, suppressing the onset of severe caries lesions, without deleterious side effects in vivo. This therapeutic approach may have broader reach as EPS are important components of matrices in most biofilms^6^ and acidic pH microenvironments can be found in other pathological conditions, such as in cystic fibrosis and *Staphylococcal* infections^28,29^. Thus, exploitation of catalytic actions by clinically approved nanomaterials could open up a new avenue for prevention of infectious diseases.

Current antimicrobial approaches, including silver nanoparticles, chlorhexidine, hydrogen peroxide, and other chemical biocides are incapable of degrading EPS and ineffective against dental caries^7,30^. Fluoride, introduced over 60 years ago, as well as more recent nanoapatites can reduce demineralization and promote remineralization but has limited antibiofilm effects^30^. We have thus discovered a topical use for ferumoxytol that can be repurposed to expand the few clinically available options for caries-preventive therapy. Immediate clinical applications of ferumoxytol-mediated catalysis could entail potentiating the efficacy of existing peroxide-based modalities, including mouthrinses and toothpastes, which contain 1.5–10% H_2_O_2_. Ferumoxytol could be locally delivered using containers with separate chambers that can keep the iron oxide nanoparticles and H_2_O_2_ separated in storage, but allowing mixing at the time of product delivery (rising or brushing). In terms of dosage, the rodent caries model has contributed to the development of clinically effective toothpastes and related caries-preventive products, including assessment of optimal fluoride concentration^26^. Thus, the currently tested topical dosage may achieve therapeutic effects clinically, although further optimization of ferumoxytol and H_2_O_2_ concentrations may be required to develop a cost-effective, safe and efficacious treatment.

Furthermore, the presence of commensal H_2_O_2_-producing organisms may provide internal substrate for ferumoxytol. The activation of locally produced H_2_O_2_ could modulate the competition between commensals and cariogenic pathogens to help maintain a healthy microbiota^14^. Conversely, *Candida albicans*, an opportunistic fungal pathogen, has been found to promote the development of plaque-biofilms associated with early childhood caries^14^. Our preliminary experiments show that such ‘ecological modulation’ concept may be tenable whereby the presence of *Streptococcus gordonii* (a commensal H_2_O_2_ producer) disrupted *S. mutans* overgrowth in biofilms treated with ferumoxytol (Supplementary Fig. 10), while also showing potential antifungal activity (Supplementary Fig. 11, *P* *<* 0.05–0.001 by paired *t* test). However, further enhancement in the catalytic performance would be required to improve the overall efficacy to kill the cariogenic pathogens. This can be achieved by taking advantage of the flexibility of iron oxide chemistry that allows the production of different nanoparticle size and shapes as well as chemical doping and surface modifications to enhance catalytic activity^5^. Importantly, inclusion of fluoride may further enhance the cariostatic effects, which could lead to improved and more effective formulations.

Finally, topical use of ferumoxytol may be particularly useful for patients with severe childhood caries, who are often linked with iron deficiency^11–13^. Recent reports from World Health Organization and Global Burden of Disease Study have pointed out that anemia caused by iron deficiency and dental caries continue to be the two most prevalent childhood health disabilities worldwide^9,10^. The possibility that major public health problems could be alleviated by the inclusion of iron oxide nanoparticle-based therapy is indeed attractive, which could lead to a therapeutic platform tailored to these severely diseased populations. Since ferumoxytol has been used off-label for treatment of pediatric patients^18^, future clinical studies to assess whether repeated topical applications of ferumoxytol, in conjunction with its systemic use, can help reduce iron deficiency and prevent severe childhood caries are worthy of exploration.

## Methods

### Characterization of ferumoxytol iron oxide nanoparticles

Ferumoxytol iron oxide nanoparticles were purchased from AMAG Pharmaceuticals (Waltham, MA). TEM was done using a Tecnai F-20 and dynamic light scattering was conducted with a Malvern Zetasizer Nanoseries (Nano ZS90) for hydrodynamic particle size determination. The thickness of carboxymethyl-dextran coating was measured by ImageJ using TEM images with 2% uranyl acetate negative stain. The peroxidase-like activity was tested via an established colorimetric assay using TMB as substrate, which generates a blue color with specific absorption at 652 nm after reacting with free-radicals catalyzed by ferumoxytol in the presence of H_2_O_2_ over time at different pH values^16^. Briefly, the reaction mixture of 500 μl 0.1 M sodium acetate (NaOAc) buffer (pH 4.5, pH 5.5, or pH 6.5) containing 20 μg ferumoxytol nanoparticles, 0.1% H_2_O_2_ (w/w) and 100 μg of TMB was incubated at room temperature and the colorimetric reaction assayed spectrophotometrically. Michaelis–Menten reaction kinetic was carried out with 20 µg ferumoxytol in 1 ml reaction buffer (0.1 M NaOAc, pH 4.5) in the presence of H_2_O_2_ at different concentrations, using 100 µg TMB as substrate. All the reactions were monitored in timescan mode at 652 nm using a Beckman DU800 spectrophotometer. Michaelis–Menten kinetic curve of ferumoxytol was acquired by plotting the respective initial velocities against H_2_O_2_ concentrations. The Michaelis constant (*Km*) were calculated via the Lineweaver–Burk plotting^16^. In a separate experiment, the catalytic activity of ferumoxytol and the leached iron ions at acidic pH were also tested. Ferumoxytol nanoparticles (500 μg) in 0.1 M NaOAc (pH 4.5) were incubated for 0, 5, 30, 60, and 120 min. After the incubation time, the free iron ions and nanoparticles were separated using ultrafiltration tubes (10 kDa, MWCO), and the iron amounts from nanoparticles and supernatant were determined by ICP-OES. The nanoparticles (resuspended in 0.1 M NaOAc buffer, pH 4.5) or supernatant were incubated with 0.5% H_2_O_2_ and 100 μg TMB, and the colorimetric reaction was also assessed as described above.

### Bacterial killing and EPS degradation by ferumoxytol activated H_2_O_2_

Time-lapse high-resolution confocal fluorescence imaging was performed to assess the dynamics of bacterial killing and glucan structure breakdown. *Streptococcus mutans* UA159 (ATCC 700610), a virulent cariogenic pathogen and well-characterized biofilm-forming strain, was grown in ultra-filtered (10-kDa cutoff; Millipore) tryptone-yeast extract (UFTYE) broth at 37 °C and 5% CO_2_ to mid-exponential phase. Ferumoxytol was added to actively growing *S. mutans* (10^8^ CFU ml^−1^) at a concentration of 1 mg ml^−1^ in the presence of 1% H_2_O_2_ at pH 4.5 or pH 6.5. SYTO 60 (652/678 nm; Molecular Probes) and propidium iodide (PI, 535/617 nm; Molecular Probes) were used for labelling live and dead cells. In addition, we conjugated ferumoxytol with Alexa Fluor 488 (490/525 nm; Molecular Probes) to visualize nanoparticle binding and localization on the cell surface. Confocal images were acquired in the same field of view at 0, 10, 20, 30, and 40 min using Zeiss LSM 800 upright single photon laser scanning microscope with a 40× (numerical aperture = 1.2) water immersion objective. Images were analyzed by ImageJ. For EPS degradation, insoluble glucans were produced by purified *S. mutans*-derived exoenzyme glucosyltransferase B (GtfB) immobilized on poly-l-lysine coated MatTek dish, and labelled with 1 μM Alexa Fluor 647-dextran conjugate (647/668 nm; Molecular Probes) as described previously^31^. The preformed fluorescently labelled glucans were then incubated with ferumoxytol (1 mg ml^−1^) and 1% H_2_O_2_ (in 0.1 M NaOAc buffer at pH 4.5 or 6.5), and time-lapsed confocal imaging was performed as described above using a 20× (numerical aperture = 1.0) water immersion objective. To further examine the glucan degradation process, we also employed computational analysis (Amira and ImageJ) that generates structural scaffold based on geometrical and topological properties of the EPS, including length and width^31^.

### In vitro biofilm model on sHA

Biofilms were formed on sHA discs (surface area = 2.7 ± 0.2 cm^2^) vertically suspended in 24-well plates using a custom-made wire disc holder, mimicking the smooth surfaces of the pellicle-coated tooth^19^ (also see Supplementary Fig. 4a). Each sHA disc was inoculated with ~2 × 10^5^ CFU of *S. mutans* per ml in UFTYE containing 1% (w/v) sucrose at 37 °C with 5% CO_2_. The culture medium was changed twice daily (at 19 and 29 h) until the end of the experimental period (43 h). The biofilms were collected and analyzed for ferumoxytol binding and catalytic activity as well as bioactivity as described below.

### Ferumoxytol binding and catalytic activity within biofilm

Quantitative assessment of ferumoxytol binding into biofilm was performed with ICP-OES. Biofilms were exposed to 2.8 ml of ferumoxytol (0.1, 0.25, 0.5, and 1 Fe mg ml^−1^) in 0.1 M NaOAc (pH 4.5) topically for 10 min at specific time-points as described in Supplementary Fig. 4a. The treated biofilms were dip-washed three times in 0.89% NaCl to remove excess and unbound agents, and then transferred to fresh culture medium. At the end of the experimental period (43 h), the biofilms were removed and homogenized by sonication (water bath sonication followed by probe sonication at an output of 7 W for 30 s; Branson Sonifier 150, Branson Ultrasonics) as detailed elsewhere^19^. The homogenized suspension was centrifuged and the biofilm pellet was washed twice with water to remove unbound material. The pellet was then dissolved in 250 μl aqua regia (HCl/HNO_3_ = 3:1) at 60 °C overnight^1,32^. The next day, 4.75 ml of MilliQ water was added and the sample was analyzed by ICP-OES (Spectro Genesis ICP) for iron content. In a separate experiment, the catalytic activity of ferumoxytol bound within intact biofilms was also assessed. Briefly, ferumoxytol or vehicle-control (buffer) treated biofilms (at 43 h) were dip-washed with 0.1 M NaOAc buffer (pH 4.5) three times and transferred to the reaction buffer 0.1 M NaOAc (pH 4.5) containing TMB and H_2_O_2_. After 30 min, the biofilms were removed and the colorimetric reaction was assessed as described above. Intact biofilms were also examined with scanning electron microscope (SEM, Quanta 600 FEG, FEI) and iron analyzed via EDS with corresponding elemental mapping on the same SEM.

We also visualized the spatial distribution of ferumoxytol, bacterial cells and the EPS-matrix using a TEM protocol optimized for high-resolution imaging of oral biofilm ultrastructure^22^. Briefly, ferumoxytol or vehicle treated biofilms (Supplementary Fig. 4a) were washed with 0.89% NaCl to remove unbound materials and treated with 2.5% glutaraldehyde in 0.89% NaCl for 2 h. Then, the biofilms were washed in 0.89% NaCl and treated with 0.05 M sodium periodate in 0.12 M NaCl for 6 h at 4 °C in the dark. The biofilms were then washed with 0.89% NaCl and reacted with 0.1 M dl-methionine in 0.12 M NaCl at room temperature overnight. The next day, the biofilms were incubated with 1% OsO_4_ for 90 min and dehydrated in a graded series of ethylene glycol/1,2-pentanediol: once 75% and 90% 1,2-pentanediol, twice 100% 1,2-pentanediol. Finally, the biofilms were treated in premixed 1:1 Epon/1,2-pentanediol for 90 min, and twice for 2 h in 100% Epon with slight agitation. The samples were then transferred to flat, polystyrene wells covered with fresh 100% Epon, and polymerized at 60 °C for 3 days. The hardened, embedded biofilms were then sectioned into ultra-thin sections (~70 nm) using a DiATOME diamond knife. Grids were poststained with 1% uranyl acetate and SATO lead staining solution. Images were taken by JEOL 1010 TEM fitted with a Hamamatsu digital camera and AMT advantage image capture software.

### Biofilm treatment and quantitative analysis

The biofilms were topically treated twice daily by placing them in 2.8 ml of ferumoxytol (1 mg ml^−1^) in 0.1 M NaOAc (pH 4.5) or vehicle-control (buffer only) for 10 min as described in Supplementary Fig. 4a. At the end of the experimental period (43 h), the ferumoxytol and vehicle treated biofilms were placed in 2.8 ml of 1% H_2_O_2_ or buffer for 5 min. After H_2_O_2_ exposure, the biofilms were removed and homogenized by sonication as described above; the sonication procedure provides optimum dispersal and maximum recoverable counts in our biofilm model without killing bacterial cells^19^. The homogenized suspension was subjected to microbiological and biochemical methods^19,27^. The total number of viable cells in each of the treated biofilms was determined by colony forming units (CFU), while insoluble extracellular polysaccharides was extracted and quantified using colorimetric assays^19,27^.

### Human derived ex vivo biofilm model on natural teeth

To further assess the antibiofilm efficacy of ferumoxytol-mediated H_2_O_2_ catalysis, we examined whether daily topical treatments can disrupt cariogenic biofilm development and prevent enamel surface damage using an ex vivo biofilm model. Plaque-biofilm samples were collected from children (age between 36 and 72 months) diagnosed with severe early childhood caries (S-ECC) as defined by the 2014 Conference Manual of the American Academy of Pediatric Dentistry. Ethical approval of the study and the written consent/permission forms were obtained from Institutional Review Board (IRB) at University of Pennsylvania (IRB 824243) prior to the study commencement. For each child, written permission form was reviewed and signed by their legal guardians. Pooled plaque samples were collected from the available smooth tooth surfaces using a sterilized periodontal scaler and transferred into 1 ml phosphate-buffered saline (PBS) in a sterilized Eppendorf tube. After collection, the plaque samples were immediately transported on ice to the laboratory, and then gently vortexed and sonicated (three 10-s pulses with 30-s intervals at 7 W) to disperse the aggregates before inoculation^33^. Different pooled samples were checked for *S. mutans*, which is frequently found in high numbers in cariogenic plaque-biofilm associated with S-ECC^34^, and total cultivable bacteria to ensure similar *S. mutans* proportion for the inoculum (Supplementary Fig. 12). The human-derived ex vivo biofilms were formed on sterilized human enamel blocks (4 mm × 4 mm) mounted vertically in 24-well plates using a custom-made wire holder (Fig. 5a). Each enamel block was inoculated with homogenized pooled plaque in UFTYE containing 1% sucrose at 37 °C and 5% CO_2_ for 115 h (5 days) as described previously^19^. To mimic topical treatment regimen, the enamel blocks and biofilms were topically treated twice-daily by placing them in 2.8 ml of ferumoxytol (1 mg ml^−1^) in 0.1 M NaOAc (pH 4.5) for 10 min immediately followed by 1% H_2_O_2_ exposure for 5 min. After each treatment, the biofilms were dip-washed with 0.89% NaCl and transferred to fresh culture medium. Biofilm were removed at 115 h for three-dimensional (3D) structural analysis and the enamel blocks were collected for surface analysis via surface topography, roughness measurement and transversal microradiography^35^.

### Analysis of ex vivo derived biofilm

The biofilms formed on enamel blocks were gently washed twice with PBS and fixed with 4% paraformaldehyde (in PBS, pH 7.4) at 4 °C for 4 h. After fixation, the biofilms were washed twice with PBS, then transferred into 50% ethanol (in PBS, pH 7.4) and stored at −20 °C. The biofilm 3D architecture was analyzed via fluorescence in situ hybridization (FISH) as detailed previously^35–37^. FISH oligonucleotide probes used in this study were: EUB338, 5′-GCTGCTCCCGTAGGATG-3′ with Cy3 for all bacteria; Smu587, 5′-ACTCCAGACTTTCCTGAC-3′ with Alexa Fluor 488 for *S. mutans*. The sample in the hybridization buffer (30% formamide, 0.9 M NaCl, 0.01% sodium-dodecyl sulphate (SDS), 20 mM Tris-HCl, pH 7.2) with the probes was incubated at 46 °C for 2 h. After incubation, the hybridized cells were washed with washing buffer (0.2 M NaCl, 20 mM Tris-HCl (pH 7.5) and 5 mM EDTA, 0.01% SDS), and further incubated at 46 °C for 10 min^35–37^. The EPS were labeled with 1 μM Alexa Fluor 647-dextran conjugate (647/668 nm; Molecular Probes)^19^. The 3D biofilm architecture was acquired using Zeiss LSM 800 with a 20× (numerical aperture =1.0) water immersion objective. The biofilms were sequentially scanned using diode lasers (488, 561, and 640 nm), and the fluorescence emitted was collected with GaAsP or multialkali PMT detector (475–525 nm for Alexa Fluor 488, 540–580 nm for Cy3, and 645–680 nm for Alexa Fluor 647-dextran conjugates, respectively. Amira 5.4.1 software (Visage Imaging) was used to create 3D renderings to visualize the architecture of the biofilms.

### Enamel surface analyses

The surface topography and roughness of the tooth enamel surface (after biofilm removal) were analyzed by a nondestructive confocal contrasting method using Zeiss LSM 800 with a C Epiplan-Apochromat 50× (numerical aperture = 0.95) nonimmersion objective. The images were processed using ConfoMap (Zeiss) to create 3D topography rendering and measure the surfaces properties in 3D. After surface analyses, enamel blocks were mounted on plastic rods and sectioned with a hard tissue microtome (Silverstone-Taylor Hard Tissue Microtome, Series 1000 Deluxe) for transversal microradiography. One 100-μm section was obtained from the center of each specimen, mounted on X-ray sensitive plates (Microchrome Technology) and subjected to X-ray, along with an aluminum step wedge. Microradiographic images were analyzed with Inspektor TMR 2000 software (ver. 1.25) with sound enamel defined at 87% mineral volume to obtain mean lesion depth (µm)^35^.

### In vivo rodent model of severe childhood caries

The therapeutic efficacy of ferumoxytol-mediated H_2_O_2_ catalysis were assessed on a well-established rodent caries model as detailed elsewhere^26,27^. Briefly, 15 days-old female Sprague–Dawley rat pups were purchased with their dams from Harlan Laboratories (Madison). Upon arrival, animals were screened for *S. mutans* and were determined not to be infected with the pathogen by plating oral swabs on mitis salivarius agar plus bacitracin. The animals were then infected by mouth with actively growing (midlogarithmic) culture of *S. mutans* UA159, and their infections were confirmed at 21 days via oral swabbing. To simulate clinical situation, we developed a combination therapy consisting of 1 min topical treatment of ferumoxytol at 1 mg ml^−1^ (or buffer) immediately followed by 1% H_2_O_2_ (or buffer) exposure. All the pups (equal numbers) were randomly placed into treatment groups, and their teeth were treated topically twice daily using a custom-made applicator (Fig. 6a)^27^. The treatment groups were: (1) control (0.1 M NaOAc buffer, pH 4.5), (2) ferumoxytol alone (1 mg ml^−1^), (3) 1% H_2_O_2_ only, and (4) ferumoxytol/H_2_O_2_. The treatments were blinded by placing the test agents in color-coded vials. Each group was provided the National Institutes of Health cariogenic diet 2000 and 5% sucrose water ad libitum. The experiment proceeded for 3 weeks (21 days). All animals were weighed weekly, and their physical appearances were noted daily. At the end of the experimental period, the animals were sacrificed, and the jaws were surgically removed and aseptically dissected, followed by sonication to recover total oral microbiota^38^. All jaws were defleshed and the teeth were prepared for caries scoring according to Larson’s modification of Keyes’ system^26,27^. Determination of caries score of the jaws was performed by a calibrated examiner who was blind for the study by using codified samples. Furthermore, both gingival and palatal tissues were collected and processed for H&E staining for histopathological analysis by an oral pathologist at Penn Oral Pathology. This study was reviewed and approved by the University of Pennsylvania Institutional Animal Care and Use Committee (IACUC #805529).

### 16S rRNA gene amplicon sequencing

Dispersed oral microbiota samples were eluted in PBS with cell lysis buffer from a DNeasy kit (Qiagen) as described by the manufacturer. After a 60 s vortex, DNA present in the buffer was isolated with the DNeasy PowerSoil HTP kit and quantitated with a spectrophotometer (Tecan). The 27F/338R primer with Golay-barcode in the reverse primer was used to amplify the V1–V2 region of 16S ribosomal DNA (16S rDNA; IDT). Four replicate PCR reactions were performed for each sample using Q5 Hot Start High Fidelity DNA Polymerase (New England BioLabs). Each PCR reaction contained: 4.3 µl microbial DNA-free water, 5 µl 5× buffer, 0.5 µl dNTPs (10 mM), 0.17 µl Q5 Hot Start Polymerase, 6.25 µl each primer (2 µM), and 2.5 µl DNA. PCR reactions without template or with synthetic DNAs were performed as negative and positive controls, respectively. PCR amplification was done on a Mastercycler Nexus Gradient (Eppendorf) using the following conditions: DNA denaturation at 98 °C for 1 min, then 20 cycles of denaturation 98 °C for 10 s, annealing 56 °C for 20 s and extension 72 °C for 20 s, last extension at 72 °C for 8 min. PCR replicates were pooled and then purified using a 1:1 ratio of Agencourt AMPure XP beads (Beckman Coulter), following the manufacturer’s protocol. The final library was prepared by pooling 10 µg of amplified DNA per sample. Those that did not reach at the DNA concentration threshold (e.g., negative control samples) were incorporated to the final pool by adding 12 µl. The library was sequenced to obtain 2 × 250 bp paired-end reads using the MiSeq Illumina^39^. Sequence data was analyzed with the QIIME pipeline (ver. 1.9.1)^40^. The forward and reverse reads were joined with no mismatches permitted. Read quality lower than Q29 or more than 3 consecutive low-quality base calls were discarded. Sequences were clustered into operational taxonomic units (OTU) at a 97% similarity threshold using the UCLUST method^41^. Taxonomic assignments were obtained based on GreenGenes 16S rRNA gene database^42^. To test the differences between communities, library vegan, and Unifrac distances were used^43,44^. Diversity, richness, and bacterial taxon abundances were compared using the Wilcoxon rank sum test. PCoA was performed using library APE for R programming language^45^.

### Statistical analysis

All the results are presented as means ± s.d. Data were analyzed using one-way analysis of variance (ANOVA) with post-hoc Tukey HSD test for multiple comparison. A pairwise comparison was conducted using Student’s *t* test. Differences between groups were considered statistically significant when *P* < 0.05. Statistical analyses were performed using SPSS version 18.0 software.

### Ethics statement

The animal experiment was conducted in strict accordance with the guidelines of the Animal Welfare Act of the United States, under the protocol reviewed and approved by the Institutional Animal Care and Use Committee of the University of Pennsylvania (IACUC#805529). The plaque-biofilm samples collection from ECC children were approved by Institutional Review Board (IRB) at University of Pennsylvania (IRB 824243) and were used only for the sole purpose of biofilm formation on enamel surfaces. The written permission form for each child was reviewed and signed by their legal guardians. The whole saliva is a convenient sample (with no identifiers) collected for the sole purpose of coating the hydroxyapatite discs for the in vitro biofilm studies. All adult subjects provided written informed consent (no children participated in the saliva collection) under the protocol reviewed and approved by the University of Pennsylvania Research Subject committee (IRB#818549).

### Data availability

The 16S rRNA gene sequences are available in the NCBI sequence read archive under accession code SRP136459. All the other data that support the findings of this study are available within the paper and its Supplementary Information Files.

## Electronic supplementary material

Supplementary Information

## References

[1] Cormode DP (2013). Inorganic nanocrystals as contrast agents in MRI: synthesis, coating and introduction of multifunctionality. NMR Biomed..

[2] Bashir MR, Bhatti L, Marin D, Nelson RC (2015). Emerging applications for ferumoxytol as a contrast agent in MRI. J. Magn. Reson. Imaging.

[3] Schwenk MH (2010). Ferumoxytol: a new intravenous iron preparation for the treatment of iron deficiency anemia in patients with chronic kidney disease. Pharmacotherapy.

[4] Zanganeh S (2016). Iron oxide nanoparticles inhibit tumour growth by inducing pro-inflammatory macrophage polarization in tumour tissues. Nat. Nanotechnol..

[5] Cormode DP, Gao L, Koo H (2018). Emerging biomedical applications of enzyme-like catalytic nanomaterials. Trends Biotechnol..

[6] Flemming H (2016). Biofilms: an emergent form of bacterial life. Nat. Rev. Microbiol..

[7] Koo H (2017). Targeting microbial biofilms: current and prospective therapeutic strategies. Nat. Rev. Microbiol..

[8] Pitts NB (2017). Dental caries. Nat. Rev. Dis. Prim..

[9] Kassebaum N (2017). Global, regional, and national prevalence, incidence, and disability-adjusted life years for oral conditions for 195 countries, 1990–2015: a systematic analysis for the global burden of diseases, injuries, and risk factors. J. Dent. Res..

[10] Vos T (2016). Global, regional, and national incidence, prevalence, and years lived with disability for 310 diseases and injuries, 1990–2015: a systematic analysis for the global burden of disease study 2015. Lancet.

[11] Clarke M (2006). Malnourishment in a population of young children with severe early childhood caries. Pediatr. Dent..

[12] Shaoul R (2012). The association of childhood iron deficiency anaemia with severe dental caries. Acta Paediatr..

[13] Schroth RJ (2013). Association between iron status, iron deficiency anaemia, and severe early childhood caries: a case–control study. BMC Pediatr..

[14] Bowen WH, Burne RA, Wu H, Koo H (2018). Oral biofilms: pathogens, matrix, and polymicrobial interactions in microenvironments. Trends Microbiol..

[15] Wei H, Wang E (2013). Nanomaterials with enzyme-like characteristics (nanozymes): next-generation artificial enzymes. Chem. Soc. Rev..

[16] Gao L (2007). Intrinsic peroxidase-like activity of ferromagnetic nanoparticles. Nat. Nanotechnol..

[17] Gao L (2014). Ferromagnetic nanoparticles with peroxidase-like activity enhance the cleavage of biological macromolecules for biofilm elimination. Nanoscale.

[18] Hassan N (2017). Intravenous ferumoxytol in pediatric patients with iron deficiency anemia. Ann. Pharmacother..

[19] Xiao J (2012). The exopolysaccharide matrix modulates the interaction between 3D architecture and virulence of a mixed-species oral biofilm. PLoS Pathog..

[20] Bowen WH (2013). The Stephan curve revisited. Odontology.

[21] Fejerskov O, Scheie AA, Manji F (1992). The effect of sucrose on plaque pH in the primary and permanent dentition of caries-inactive and -active Kenyan children. J. Dent. Res..

[22] Reese S, Guggenheim B (2007). A novel TEM contrasting technique for extracellular polysaccharides in in vitro biofilms. Microsc. Res. Tech..

[23] Spiteller G (2003). Are lipid peroxidation processes induced by changes in the cell wall structure and how are these processes connected with diseases?. Med. Hypotheses.

[24] Halliwell B (1987). Oxidants and human disease: some new concepts. FASEB J..

[25] Liu Y (2016). Topical delivery of low-cost protein drug candidates made in chloroplasts for biofilm disruption and uptake by oral epithelial cells. Biomaterials.

[26] Bowen WH (2013). Rodent model in caries research. Odontology.

[27] Horev B (2015). pH-activated nanoparticles for controlled topical delivery of farnesol to disrupt oral biofilm virulence. ACS Nano..

[28] Mercier RC, Stumpo C, Rybak MJ (2002). Effect of growth phase and pH on the in vitro activity of a new glycopeptide, oritavancin (LY333328), against *Staphylococcus aureus* and *Enterococcus faecium*. J. Antimicrob. Chemother..

[29] Poschet J, Perkett E, Deretic V (2002). Hyperacidification in cystic fibrosis: links with lung disease and new prospects for treatment. Trends Mol. Med..

[30] Featherstone JD, Doméjean S (2012). The role of remineralizing and anticaries agents in caries management. Adv. Dent. Res..

[31] Hwang G, Koltisko B, Jin X, Koo H (2017). Nonleachable imidazolium-incorporated composite for disruption of bacterial clustering, exopolysaccharide-matrix assembly, and enhanced biofilm removal. ACS Appl. Mater. Interfaces.

[32] Naha PC (2014). Dextran coated bismuth–iron oxide nanohybrid contrast agents for computed tomography and magnetic resonance imaging. J. Mater. Chem. B.

[33] Xiao J (2016). *Candida albicans* carriage in children with severe early childhood caries (S-ECC) and maternal relatedness. PLoS One.

[34] Hajishengallis E, Parsaei Y, Klein MI, Koo H (2017). Advances in the microbial etiology and pathogenesis of early childhood caries. Mol. Oral. Microbiol..

[35] Xiao J (2017). Biofilm three-dimensional architecture influences in situ pH distribution pattern on the human enamel surface. Int. J. Oral. Sci..

[36] Thurnheer T, Gmür R, Guggenheim B (2004). Multiplex FISH analysis of a six-species bacterial biofilm. J. Microbiol. Methods.

[37] Kim D, Sitepu IR, Hashidoko Y (2013). Induction of biofilm formation in the betaproteobacterium *Burkholderia unamae* CK43B exposed to exogenous indole and gallic acid. Appl. Environ. Microbiol..

[38] Klein MI (2012). Molecular approaches for viable bacterial population and transcriptional analyses in a rodent model of dental caries. Mol. Oral. Microbiol..

[39] Caporaso JG (2012). Ultra-high-throughput microbial community analysis on the illumina HiSeq and MiSeq platforms. ISME J..

[40] Caporaso JG (2010). QIIME allows analysis of high-throughput community sequencing data. Nat. Methods.

[41] Edgar RC (2010). Search and clustering orders of magnitude faster than BLAST. Bioinformatics.

[42] McDonald D (2012). An improved Greengenes taxonomy with explicit ranks for ecological and evolutionary analyses of bacteria and archaea. ISME J..

[43] Oksanen, J., Blanchet, F.G. & Kindt, R. Vegan: community 880 ecology package. R package version 2.3-0. https://cran.r-project.org/web/packages/vegan/index.html

[44] Lozupone C, Knight R (2005). UniFrac: a new phylogenetic method for comparing microbial communities. Appl. Environ. Microbiol..

[45] Paradis E, Claude J, Strimmer K (2004). APE: analyses of phylogenetics and evolution in R language. Bioinformatics.

