# Supplementary material for: Topical ferumoxytol nanoparticles disrupt biofilms and prevent tooth decay in vivo via intrinsic catalytic activity
Source: Nat Commun. 2018 Jul 31;9:2920. doi: 10.1038/s41467-018-05342-x (PMC6068184; doi:10.1038/s41467-018-05342-x)
Supplement: Supplementary file 1 — Supplementary Information [file 41467_2018_5342_MOESM1_ESM.pdf]

Supplementary Information

Topical ferumoxytol nanoparticles disrupt biofilms and prevent severe tooth decay *in vivo* via intrinsic catalytic activity

Liu et al.

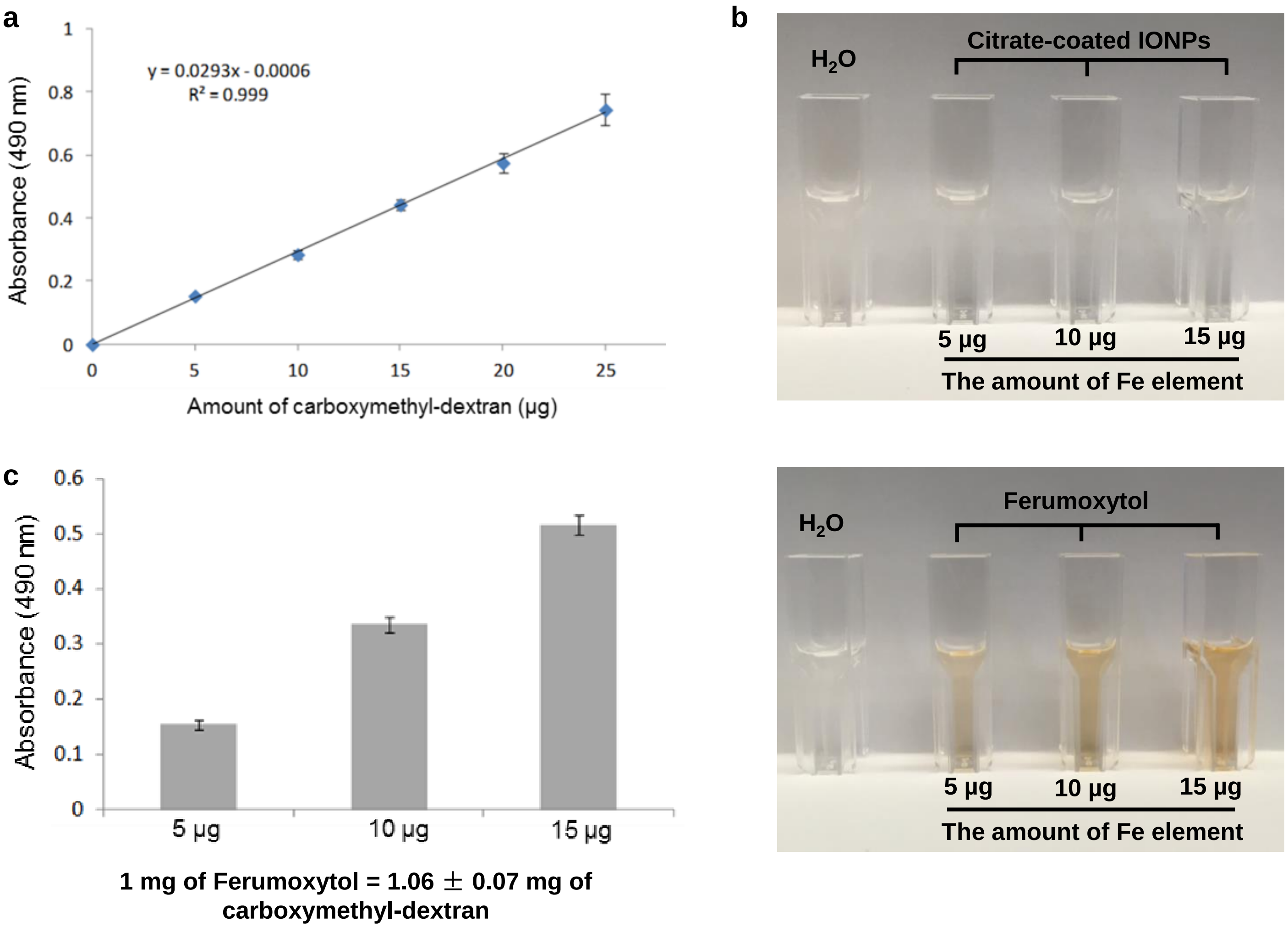

Supplementary Fig. 1. Quantification of dextran in ferumoxytol nanoparticles using a colorimetric (phenol-sulfuric) method. **a,** Standard curve using carboxymethyl-dextran. **b,** Representative images of citrate-coated iron oxide nanoparticles (IONPs) and ferumoxytol nanoparticles after colorimetric reaction. **c,** Absorbance at 490 nm of different amounts of ferumoxytol (based on Fe element). The data are presented as the mean ± s.d. from three independent assays.

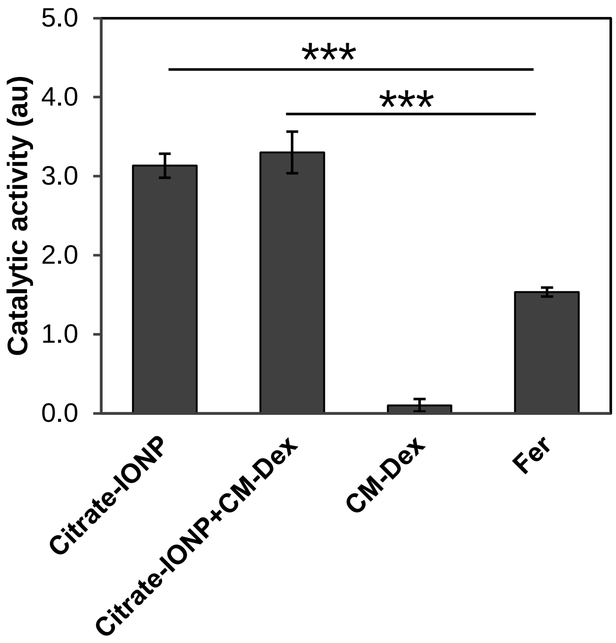

Supplementary Fig. 2. Catalytic activity of citrate-IONP (0.5 mg ml^{-1}) as determined by 3,3',5,5'-tetramethylbenzidine (TMB) method. The data are presented as the mean ± s.d. from three independent experiments (n=6). The quantitative data were subjected to Student's *t*-test for a pairwise comparison. *** $P < 0.001$. CM-Dex, carboxymethyl-dextran; Fer, ferumoxytol.

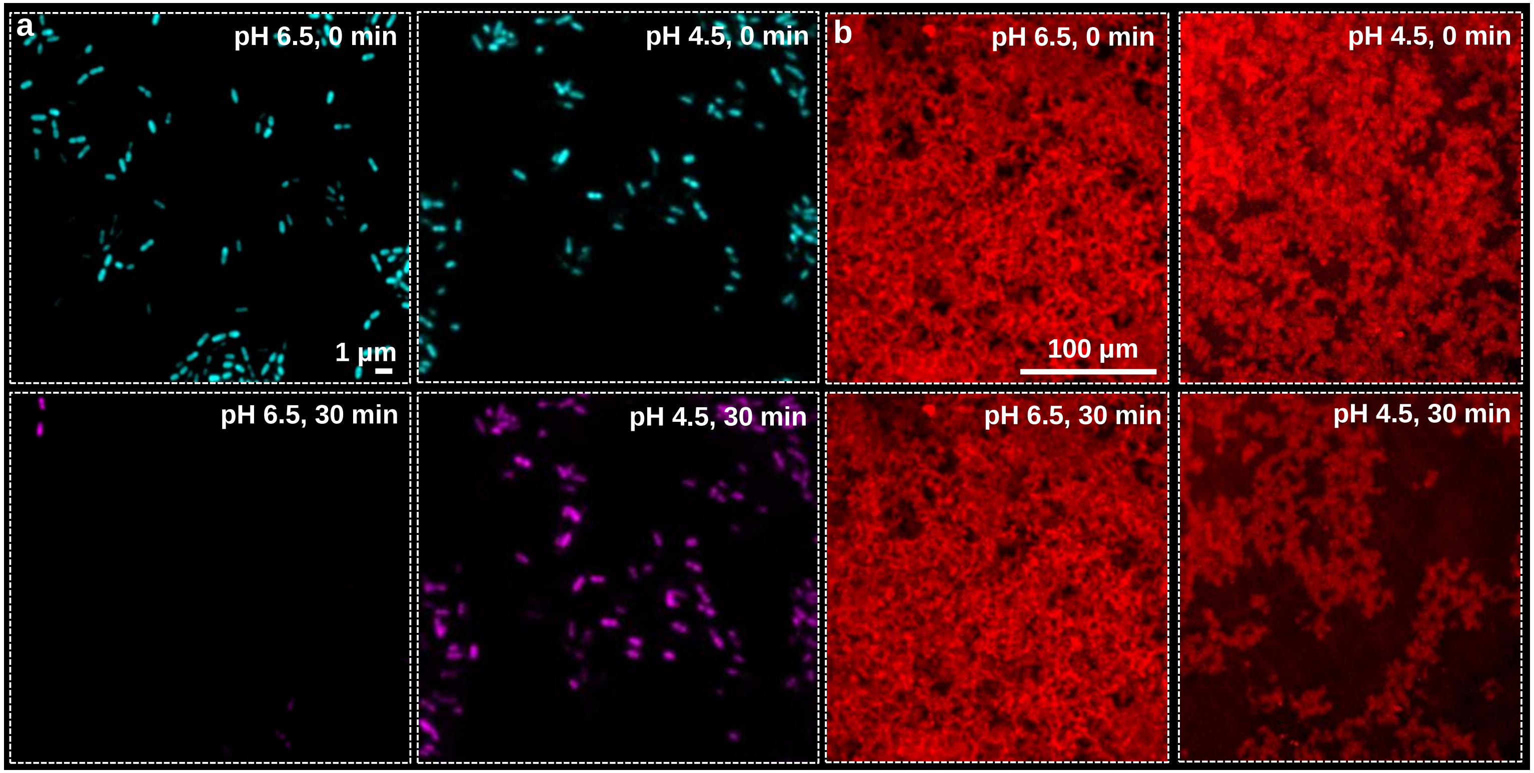

Supplementary Fig. 3. Dynamics of bacterial killing and EPS glucans degradation *in situ* by ferumoxytol-mediated H_2O_2 catalysis. **a**, Bacterial killing activity of ferumoxytol (1 mg ml^{-1}) with H_2O_2 (1%) at pH 6.5 vs pH 4.5. *S. mutans* cells were labeled by SYTO 60 (in blue) and PI stained the membrane-damaged dead cells (in purple) (n=4). **b**, Degradation of EPS (labelled with Alexa Fluor 647; in red) by ferumoxytol and H_2O_2 at different pH values (n=4).

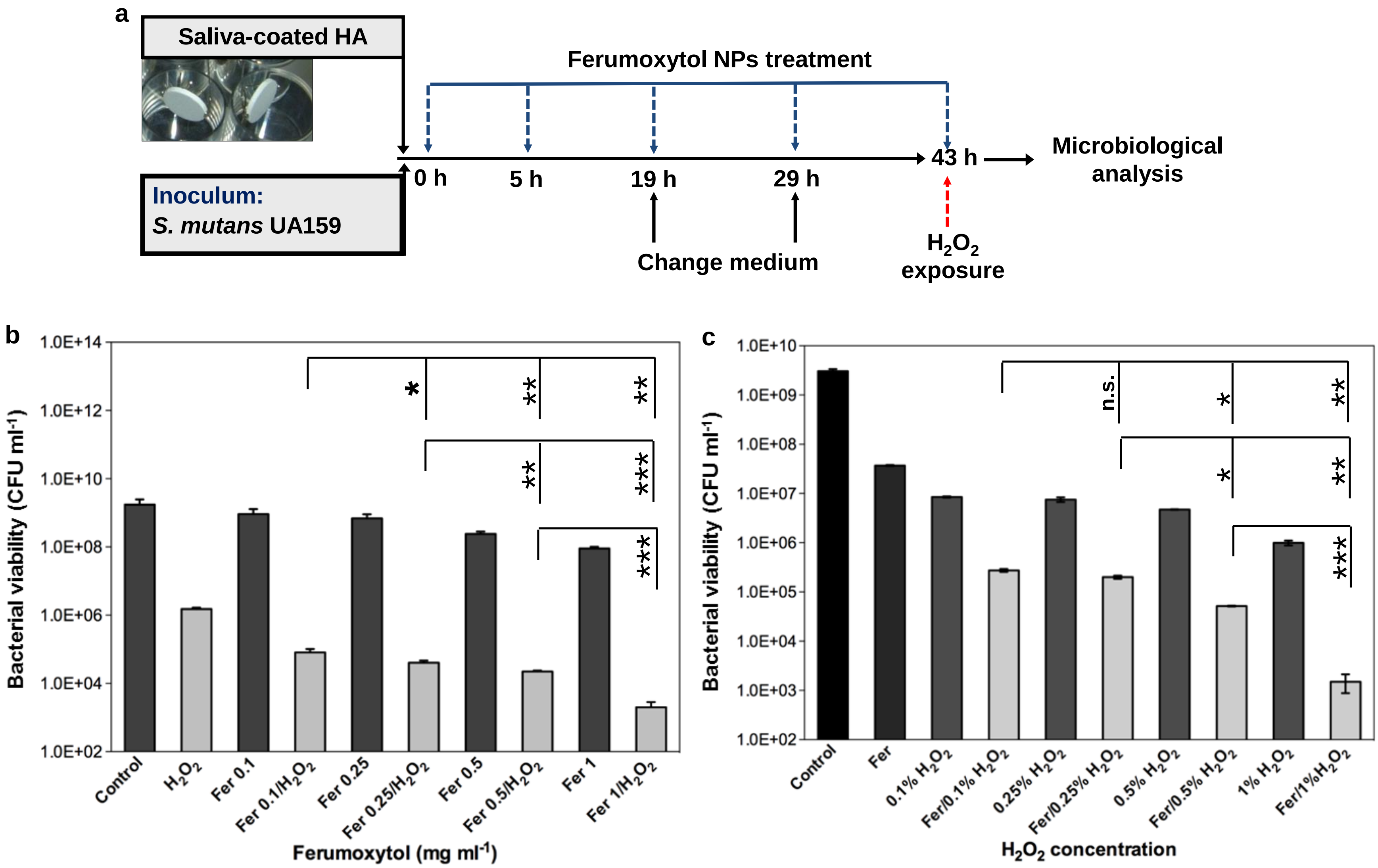

Supplementary Fig. 4. Experimental design and antibiofilm activity of ferumoxytol/H_2O_2 at different concentrations. a, Overall biofilm experimental design and topical treatment regimen. **b**, Antibacterial activity of different concentration of ferumoxytol (0.1, 0.25, 0.5, 1 mg ml^{-1}) with H_2O_2 at 1% as determined by total viable cells counting (colony forming units, CFU). **c**, Antibacterial activity of ferumoxytol at 1 mg ml^{-1} with different concentration of H_2O_2 (0.1, 0.25, 0.5, 1%). The data are presented as the mean ± s.d. (n = 4) and the quantitative data were subjected to Student's *t*-test for a pairwise comparison. * $P < 0.05$, ** $P < 0.01$, *** $P < 0.001$; n.s., non-significant. Fer, ferumoxytol.

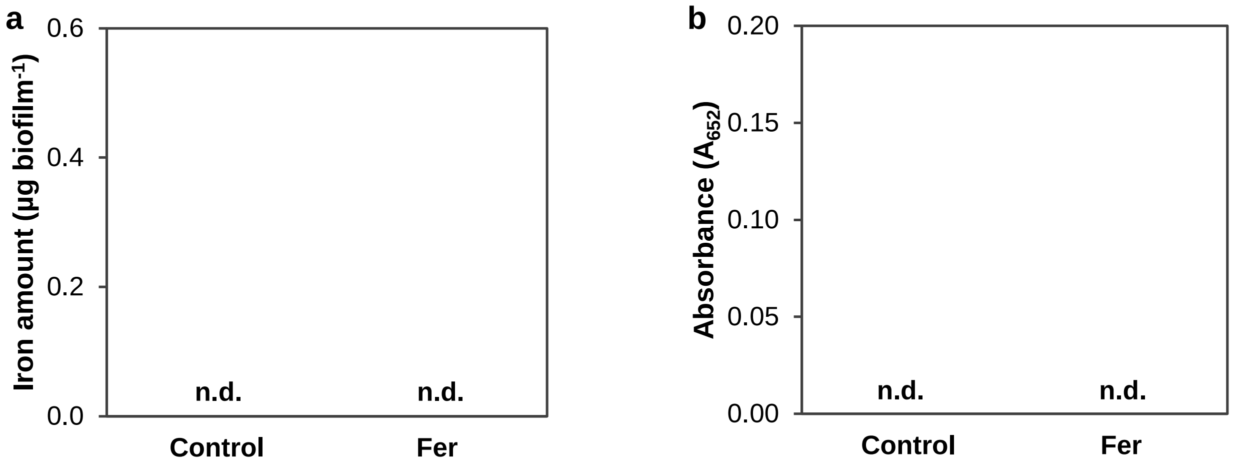

Supplementary Fig. 5. Retention and *in situ* catalytic activity of ferumoxytol in biofilms formed with glucose. *Streptococcus mutans* UA159 biofilms were formed on sHA disc surfaces with 1% glucose (w/v) and the treatment was carried out as shown in Supplementary Fig. 4a. **a**, Amount of ferumoxytol (Fer) bound via ICP-OES and (**b**) catalytic activity within biofilms as measured by 3,3',5,5'-tetramethylbenzidine (TMB) reaction. Non-detectable (n.d.) amounts of iron and catalytic activity were found from three independent experiments (n=6).

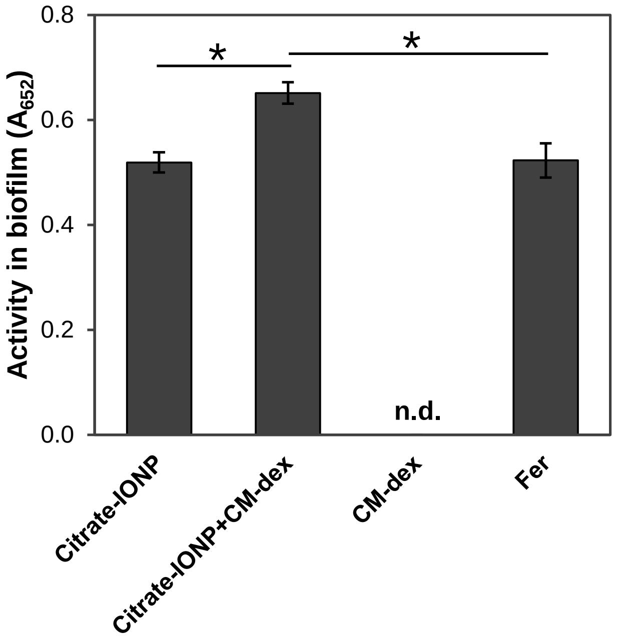

Supplementary Fig. 6. Catalytic activity within biofilms. *S. mutans* biofilms were formed on sHA disc surfaces and the treatment was carried out as shown in Supplementary Fig. 4a. The concentration of citrate-coated IONPs or ferumoxytol was 0.5 mg ml^{-1}. The data are presented as the mean ± s.d. from three independent experiments (n=6). The quantitative data were subjected to Student's *t*-test for a pairwise comparison. * $P < 0.05$; n.d., non-detectable. CM-Dex, carboxymethyl-dextran; Fer, ferumoxytol.

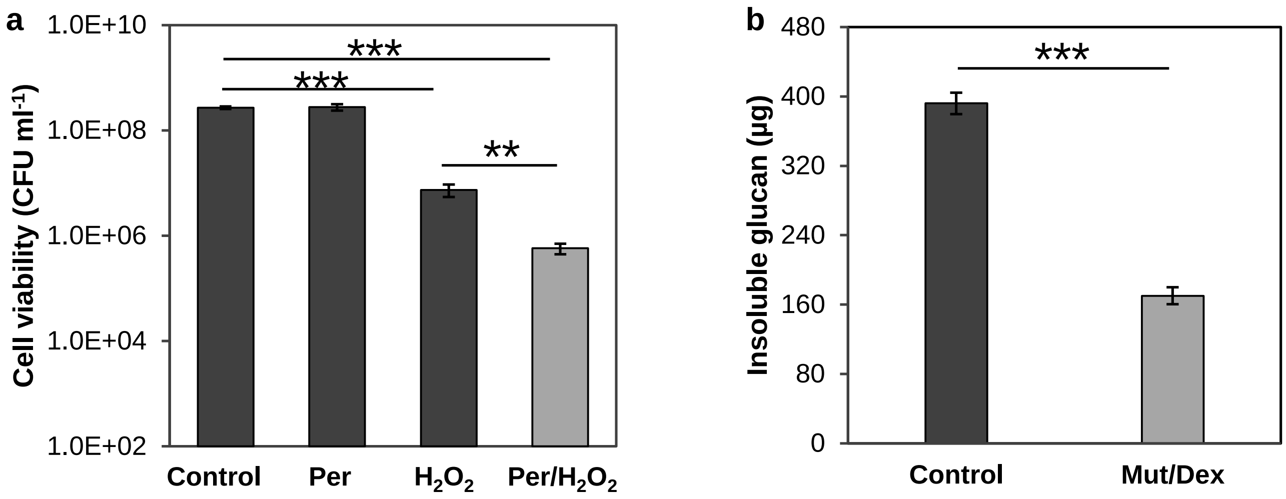

Supplementary Fig. 7. Antibacterial effect of peroxidase (Per) and EPS degradation by glucanohydrolases (Mut/Dex). **a**, *S. mutans* was grown in UFYTE broth containing 1% glucose at 37˚C and 5% CO_2 to mid-exponential phase. Peroxidase was added to actively growing *S. mutans* (10^8 CFU ml^{-1}) at a concentration of 20 ng ml^{-1} in the presence of 1% H_2O_2. The number of viable cells (total number of colony forming units, CFU) was determined; **b**, Insoluble glucans were preformed by glucosyltransferase B (GtfB) on MatTek dish. Glucanohydrolases, mutanase and dextranase (Mut/Dex, 5 units/5 units), were used to digest EPS glucans. The data are presented as the mean ± s.d. from two independent experiments (n=4). The quantitative data were subjected to Student's *t*-test for a pairwise comparison. ** $P < 0.01$, *** $P < 0.001$.

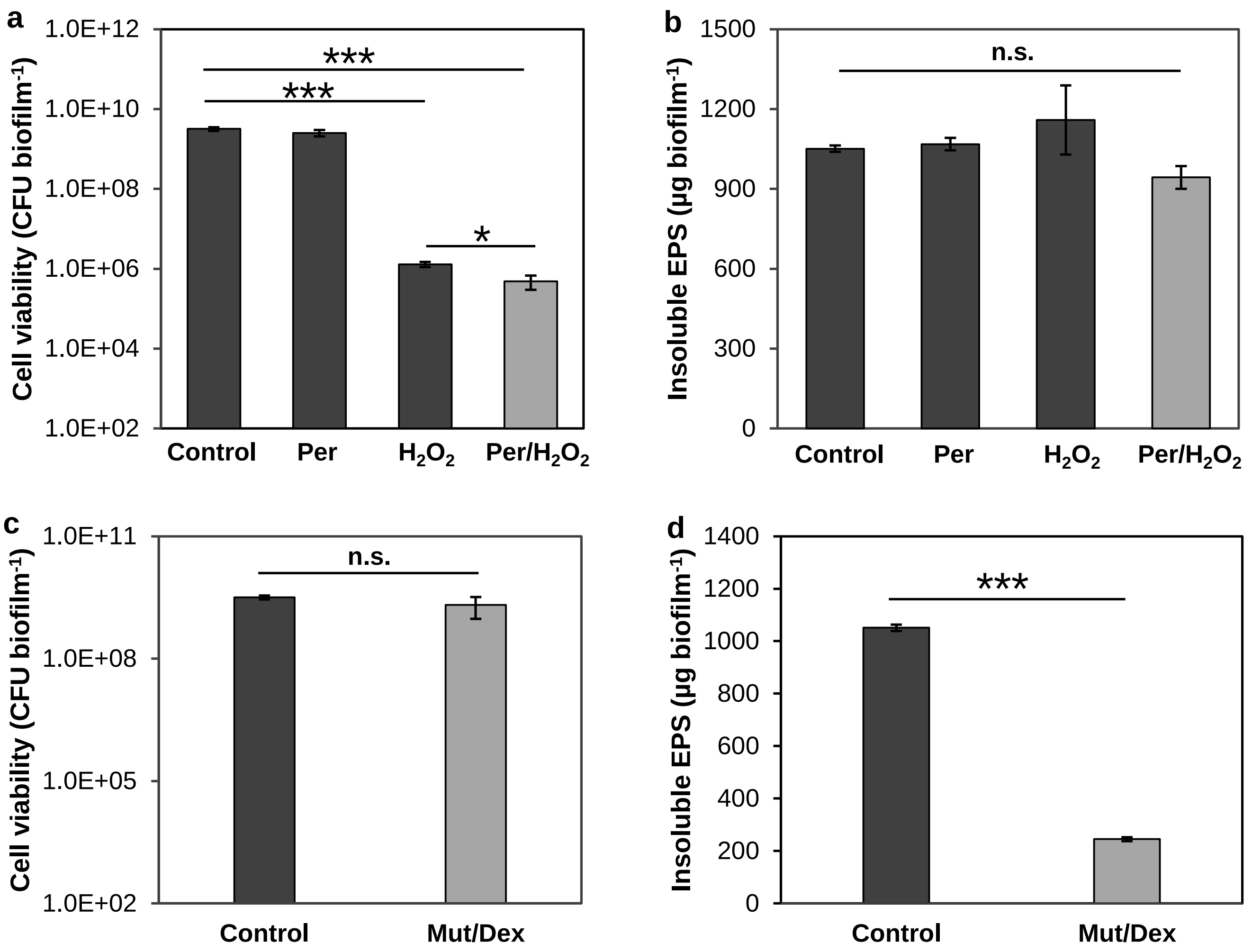

Supplementary Fig. 8. Antibiofilm effect of peroxidase (Per) and glucanohydrolases (Mut/Dex). a and **b.** Effect on the viability of *S. mutans* cells within biofilms as well as EPS degradation following exposure to peroxidase (20 ng ml^{-1}) and H_2O_2 (1%); **c** and **d.** The viability of *S. mutans* cells in biofilms and EPS degradation after mutanase/dextranase (25 units/5 units) treatment. The data are presented as the mean ± s.d. from two independent experiments (n=4). The quantitative data were subjected to Student's *t*-test for a pairwise comparison. * $P < 0.05$, *** $P < 0.001$; n.s., non-significant.

a

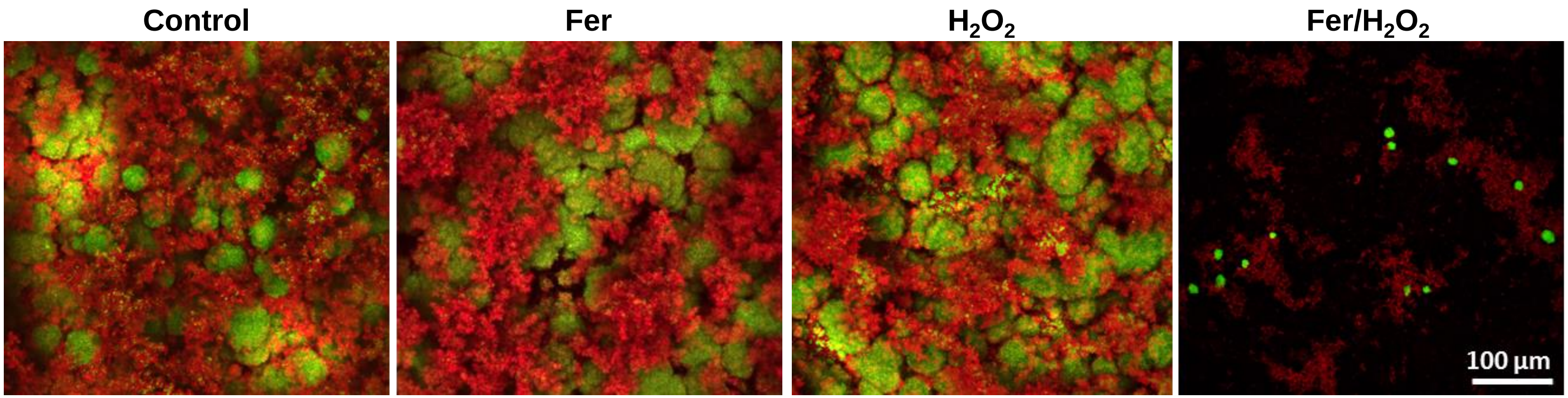

b

COMSTAT analysis

Biofilm 43 h	Total biovolume (μm³/μm²)	Cell biovolume (μm³/μm²)	EPS biovolume (μm³/μm²)
Control	225.1±12.1	86.3±6.8	138.7±11.5
Fer	231.9±10.3	87.5±5.5	144.5±7.1
H_2O_2	230.0±15.1	89.5±4.4	140.4±11.3
Fer/H_2O_2	22.2±4.2***	5.4±1.1***	16.7±3.3***

Supplementary Fig. 9. Biofilm disruption after topical treatment with ferumoxytol and H_2O_2 alone or in combination. a, Representative confocal images of biofilms treated with ferumoxytol (Fer) and/or H_2O_2. Biofilms were topically treated by ferumoxytol then immediately followed by H_2O_2 exposure (Fer/H_2O_2) or sodium acetate buffer (ferumoxytol alone; Fer) twice daily. For H_2O_2 alone, biofilms were treated with sodium acetate buffer then immediately followed by H_2O_2 exposure. The control group consisted of biofilms treated with buffer only. Topical treatments twice-daily were performed, and the treated biofilms analyzed at 43h. Bacterial cells were stained with SYTO 9 (in green) and EPS were labelled with Alexa Fluor 647 (in red). **b,** Quantitative analysis of bacteria and EPS biovolume within each treated biofilm was done using COMSTAT. Data are shown as mean ± s.d. (n=6). The quantitative data were subjected to Student's *t*-test for a pairwise comparison. *** $P < 0.001$ (vs. control).

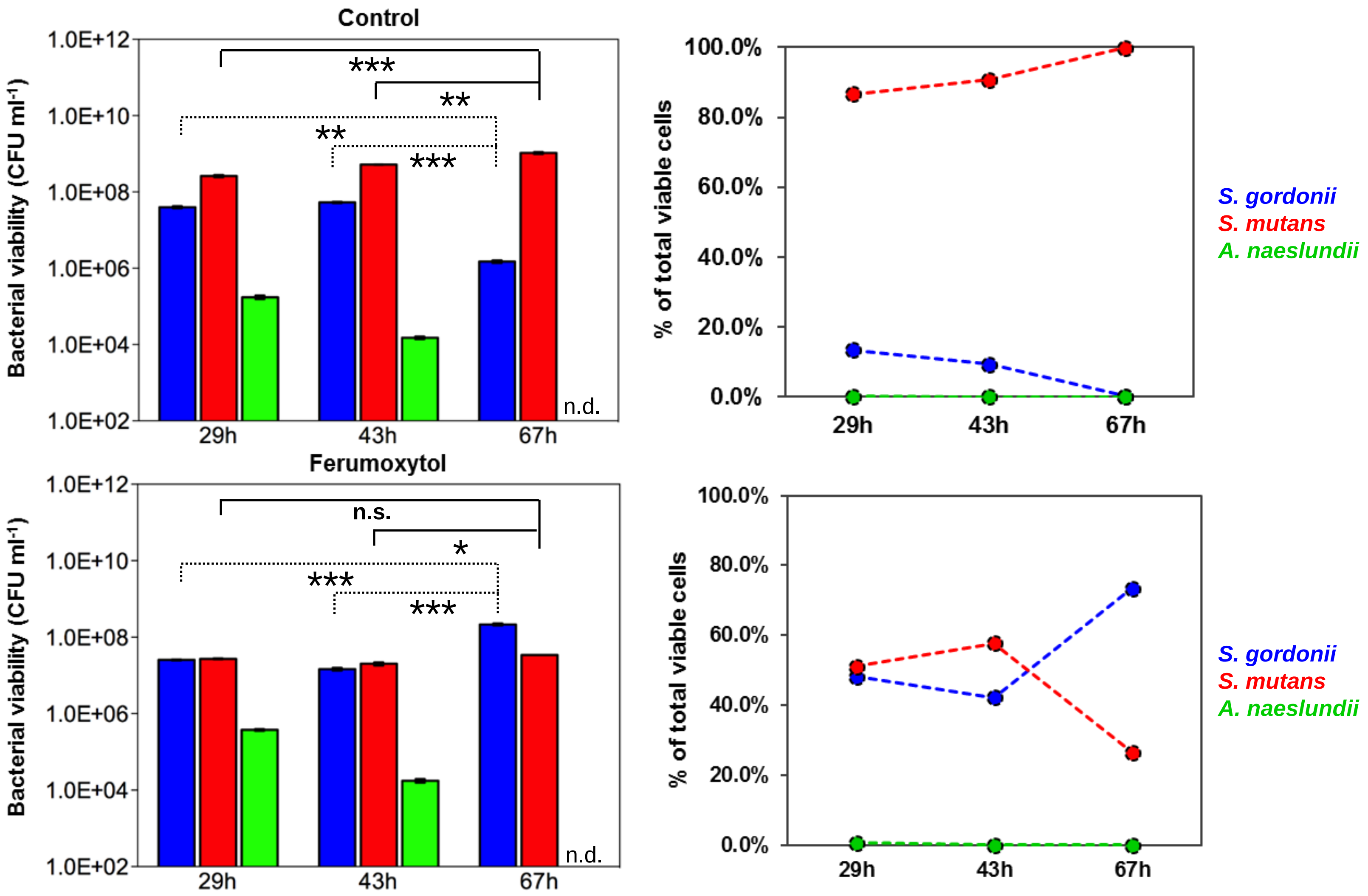

Supplementary Fig. 10. Dynamic changes of microbial composition of mixed-species biofilms with or without topical ferumoxytol exposure. Mixed-species biofilms were formed on saliva-coated hydroxyapatite discs. A defined microbial population of *Streptococcus mutans* (10^4 CFU ml^{-1}), *Streptococcus gordonii* (10^4 CFU ml^{-1}), and *Actinomyces naeslundii* (10^6 CFU ml^{-1}) was inoculated in 2.8 ml of UFTYE containing 0.1% (w/v) sucrose (0 h) and cultured without disturbance to form an initial biofilm community on the sHA surface. At 29h, the biofilms were transferred to UFTYE containing 1% sucrose to induce environmental changes simulating a cariogenic challenge. The culture medium was changed twice daily (8 a.m. and 6 p.m.) until the end of the experimental period (67h). Biofilm treatments started at 5 h of Day 1 and were carried out at 8 a.m. and 6 p.m. each day. Total viable cells for each experimental group are shown in the left graphs, while the proportion of different bacterial species in the treated biofilms is presented in the right graphs (n = 4). In control group, *S. mutans* was the dominant species from 29 h to the endpoint, while the viable populations of *S. gordonii* and *A. naeslundii* declined. In contrast, ferumoxytol-treated biofilms (1 mg ml^{-1}) were characterized by *S. gordonii* dominance at 67 h without causing significant changes in the *S. mutans* viable population. The data presented as mean ± s.d., and one-way ANOVA with post-hoc Tukey HSD test was used for a multiple comparison. * $P < 0.05$, ** $P < 0.01$, *** $P < 0.001$; n.d., non-detectable; n.s., non-significant.

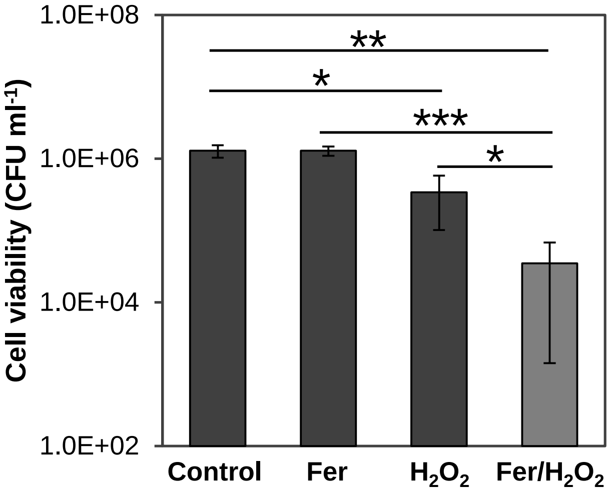

Supplementary Fig. 11. Anti-fungal effects of ferumoxytol-mediated H_2O_2 catalysis. *Candida albicans* SC 5314 was grown in ultra-filtered tryptone-yeast extract (UFYTE) broth at 37˚C and 5% CO_2 to mid-exponential phase. Ferumoxytol was added to actively growing *C. albicans* (10^6 CFU ml^{-1}) at a concentration of 1 mg ml^{-1} in the presence of 1% H_2O_2. The number of viable cells (total number of colony forming units, CFU) was determined. The data are presented as the mean ± s.d. from three independent experiments (n=6). The quantitative data were subjected to Student's *t*-test for a pairwise comparison. * $P < 0.05$, ** $P < 0.01$, *** $P < 0.001$.

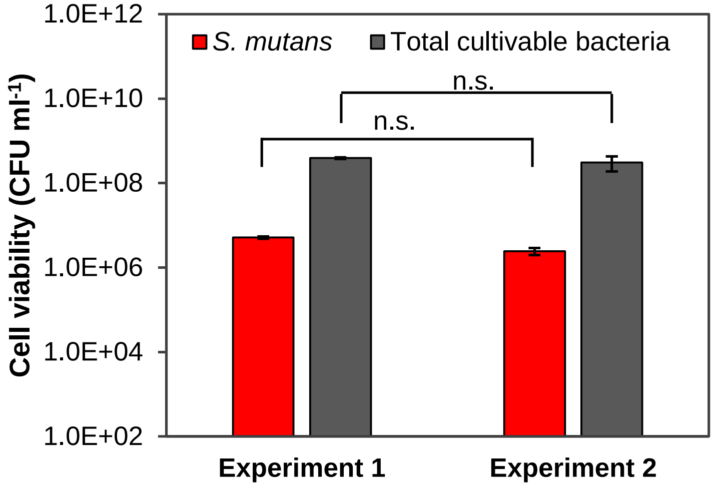

Supplementary Fig. 12. The numbers of total bacteria and *S. mutans* viable population in pooled-plaque samples for *ex vivo* biofilm experiments. Different pooled samples were checked for *S. mutans* and total cultivable bacteria to ensure similar *S. mutans* proportion for the inoculum. The data are presented as the mean ± s.d. and the quantitative data were subjected to Student's *t*-test for a pairwise comparison. n.s., non-significant.
